# Learning motifs and their hierarchies in atomic resolution microscopy

**DOI:** 10.1126/sciadv.abk1005

**Published:** 2022-04-13

**Authors:** Jiadong Dan, Xiaoxu Zhao, Shoucong Ning, Jiong Lu, Kian Ping Loh, Qian He, N. Duane Loh, Stephen J. Pennycook

**Affiliations:** 1 NUS Graduate School for Integrative Sciences and Engineering, National University of Singapore, 21 Lower Kent Ridge, Singapore 119077, Singapore.; 2 Department of Materials Science and Engineering, National University of Singapore, 9 Engineering Drive 1, Singapore 117575, Singapore.; 3 NUS Centre for Bioimaging Sciences, National University of Singapore, 14 Science Drive 4, Singapore 117557, Singapore.; 4 School of Materials Science and Engineering, Nanyang Technological University, Singapore 639798, Singapore.; 5 Department of Chemistry, National University of Singapore, 3 Science Drive 3, Singapore 117543, Singapore.; 6 Department of Physics, National University of Singapore, 2 Science Drive 3, Singapore 117551, Singapore.; 7 Department of Biological Sciences, National University of Singapore, 16 Science Drive 4, Singapore 117558, Singapore.

## Abstract

Characterizing materials to atomic resolution and first-principles structure-property prediction are two pillars for accelerating functional materials discovery. However, we are still lacking a rapid, noise-robust framework to extract multilevel atomic structural motifs from complex materials to complement, inform, and guide our first-principles models. Here, we present a machine learning framework that rapidly extracts a hierarchy of complex structural motifs from atomically resolved images. We demonstrate how such motif hierarchies can rapidly reconstruct specimens with various defects. Abstracting complex specimens with simplified motifs enabled us to discover a previously unidentified structure in a Mo─V─Te─Nb polyoxometalate (POM) and quantify the relative disorder in a twisted bilayer MoS_2_. In addition, these motif hierarchies provide statistically grounded clues about the favored and frustrated pathways during self-assembly. The motifs and their hierarchies in our framework coarse-grain disorder in a manner that allows us to understand a much broader range of multiscale samples with functional imperfections and nontrivial topological phases.

## Teaser

An interpretable machine learning framework learns structural motifs and their compositional hierarchies in complex materials.

## INTRODUCTION

Physical systems are often modeled as an ensemble of recurring motifs. These include atomic structural features within periodic unit cells, atomic point defects (*1*–*3*), extended line defects (*4*–*6*), topological phases (*7*–*10*), and superlattices (*11*–*13*). The composition and spatial arrangement of these motifs underpin the physics of materials. In these cases, rationally inspired discovery of novel phases and structures using first-principles approaches can be impractical, especially when they contain extended nonperiodic features. Such examples are abundant in the rapidly developing field of twistronics, where twisting two monolayers of certain materials can make the resultant Moiré bilayer into a superconductor (*14*), Mott insulator (*15*), magnet (*16*), or trap light as solitons (*17*). Another example is polyoxometalate (POM), which comprises structural motifs of transition metal oxyanions, that can form a diverse range of three-dimensional (3D) enabling frameworks for catalysis, memory storage, and even nanomedical applications (*18*, *19*). For such complex systems, one often turns to exploratory high-resolution microscopy to discover novel structures in the laboratory.

Automatically discovering interpretable unseen structural motifs from atomic resolution images remains an unsolved challenge. Consequently, our ability to rapidly describe novel and complex structures from high-resolution but noisy, incomplete images is severely handicapped. This, in turn, breaks the real-time feedback for efficiently seeking relevant regions in complex samples and for making timely decisions on sample characterization and preparation.

Machine learning (ML) models can be trained to recognize specific types of structural motifs (*20*–*23*) presented at a particular range of resolution, rotations, translations, and noisy imaging conditions. In many cases, training these supervised models on experimental measurements still requires a sufficiently large corpus of labeled data, which often come from laborious labeling by a human expert or idealized forward models (e.g., simulators) with ground truth. However, it is a well-known issue (*24*–*26*) that applying these ML models to slightly different samples and/or presentations can produce wrong predictions. Furthermore, while feature extraction has been automated for some atomic resolution micrographs (*27*–*29*), in some cases, these output features are abstract and not readily interpretable as structural motifs (*27*, *30*).

Clustering is an established and powerful form of unsupervised learning that does not require labels. These models rapidly yield feature classes that are readily interpretable. Furthermore, such clustering can be noise robust when feature classes are formed by signal averaging noisy and incomplete observations of potential class members. However, these unsupervised ML models do not efficiently model the spatial context of the derived features. Just like this paragraph is not merely a “bag of words” (*31*), a high-resolution sample is more than a collection of structural motifs. The spatial context surrounding each motif and the arrangement of neighboring motifs are crucial. Such spatial context gives us crucial information about how these motifs self-assemble and shed light on mechanisms that encourage or frustrate them.

For such context-aware motif learning, we are inspired by artificial deep neural networks’ (DNNs) powerful ability to model complex relationships between features: Convolutional neural networks can learn spatial relationships between nearby pixels in a hierarchical fashion (*32*, *33*), and natural language processing models can learn compositional rules of words, phrases, and fragments (*34*, *35*). However, training such supervised DNNs needs labeled data, which are typically derived from laborious manual labeling of atomic resolution micrographs. The label-free alternative using unsupervised DNNs learns features that are not optimized to be human interpretable and hence not primed for insightful codiscovery with humans.

Without using DNNs, it is possible to augment a clustering-based unsupervised classifier with the ability to learn hierarchical relationships between structural motifs. Hence, the representations learned by such a model are readily interpretable as motifs and their contextual hierarchies. Here, we adopt a classify-then-compose framework that analogously decodes the fundamental motifs (“building blocks”) of a micrograph using unsupervised learning, from which more complex motifs are hierarchically and interpretably constructed. Such a bottom-up approach can be useful for context-aware learning of unseen, complex structural motifs with minimal to no supervision. The learning objective is twofold: rapidly obtain human-interpretable features for structural motifs obtained from a noisy atomic resolution image and rapidly characterize the compositional rules of these motifs.

## RESULTS

The first step of this framework is to extract structural motifs within the sample, some of which might be previously unknown. Periodic atomic structures can be described by a finite number of atom-centered motifs (fig. S1). In realistic samples and imaging conditions, however, each of these motifs will have additional internal parameters that describe imaging uncertainties (e.g., measurement errors), strain fields, or other latent factors (figs. S2 and S3). Complex motifs can, in turn, be constructed from simpler motifs, to describe longer-range order within complex samples, and systematically create a taxonomy for classifying increasingly complex motifs.

**Fig. 1.**
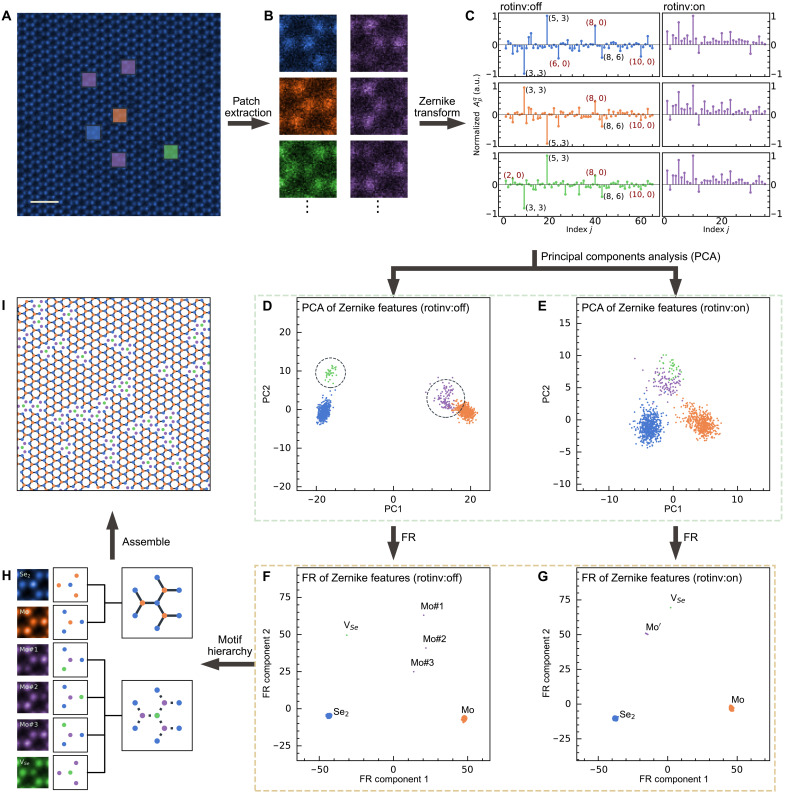
Interpretable ML workflow to construct a hierarchy of atomic structural motifs. (**A**) ADF-STEM image of monolayer MoSe_2_. Scale bar, 0.5 nm. (**B**) Automatically extracting atom-centered image patches from (A). (**C**) Computing the Zernike features within the image patches in (B) either in their rotationally invariant representation (rotinv:on) or retaining rotation information (rotinv:off). (**D**) and (**E**) project the patches’ Zernike representations in their first two principal components. (**F**) Applying an FR clustering algorithm acting on these Zernike features automatically classifies them into different atomic structural motifs; (**G**) clustering on the rotationally invariant representations (rotinv:on) neatly groups features centered on Mo atoms together. (**H**) Each cluster in (F) represents a different structural motif: patches centered on two Se (Se_2_), single Se vacancies (V_Se_), or single Mo columns that are next to vacancies (Mo#1, Mo#2, and Mo#3) and those that are not (Mo). These motifs can, in turn, be composed as two classes of atom column quartets. (**I**) Reconstruction of (A) with these atom column quartets. a.u., arbitrary units.

**Fig. 2.**
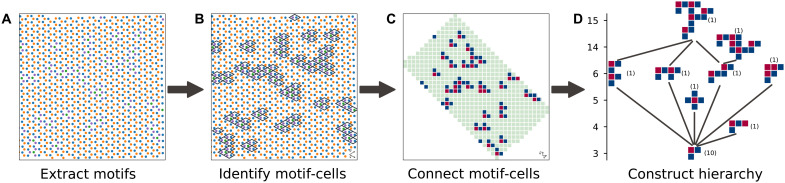
Steps in the automatic construction of hierarchy of motif-cells found in the monolayer MoSe_2_ sample. (**A**) Lowest-level motifs (represented by color dots) from Fig. 1I and their corresponding positions. (**B**) Bin motifs into motif-cells that are defined by real-space lattice vectors. All motif-cells are classified according to their motif compositions. The minority motif-cells, which correspond to two types of defects, are outlined here as rhombuses. (**C**) Mapping real-space positions of two types of minority motif-cells (blue and red) into a square grid: spatial locations of cells in (B) shown on a square grid delineated by their cells’ real-space lattice indices (rather than vectors). Connected minority motif-cells (von Neumann neighbors) are programmatically identified as higher-level motif-cells. (**D**) These motif-cells are ordered according to the number of cells that they contain (enumerated on vertical axis), where differences up to a fourfold rotation symmetry are ignored. The number of occurrences of each type of motif-cell is shown in parentheses. We associate higher-level motif-cells with lower-level ones if the spatial arrangement of cells in the latter occurs within the former; here, edges are drawn between associated motif-cells that are the nearest in the hierarchy.

We illustrate the four steps of our learning framework using the annular dark-field scanning transmission electron microscopy (ADF-STEM) image of monolayer MoSe_2_ in Fig. 1A. The first step is patch extraction, where we programmatically extract all fixed-size image patches that are centered on all the visible atom columns (Fig. 1B) by seeking regions of high local contrast or symmetry. The second step is feature extraction, where the features in these patches are projected onto the Zernike polynomial (ZP) bases (Fig. 1C). In the third step, these Zernike features are grouped into different motifs using a multistage force-relaxed (FR) clustering algorithm (Fig. 1, D to G), which we describe below (also see Materials and Methods and movie S1). In addition, last, a hierarchy of increasingly complex motifs is automatically constructed (Fig. 1H), from which the original sample is reconstructed (Fig. 1I). This hierarchy building toward more complex motif combinations is elaborated in Fig. 2.

The second step of this framework (Fig. 1C) uses ZPs to reduce the representation of the configurations and shapes of the atom columns in pixelated image patches. This is in contrast to the dimensionality reduction for motif building by Belianinov *et al.* (*28*), where only atomic column configurations were preserved. This Zernike representation offers three key advantages. First, the completeness and orthogonality of ZPs guarantee that any square-integrable function on the unit circle can be decomposed into a linear combination of ZPs with coefficients named Zernike moments without redundancy (fig. S4). In Fig. 1C, these Zernike moments ($A_{p}^{q}$) are uniquely indexed by either the (*p, q*) tuple or using the equivalent single index *j* (table S1). Second, ZP decomposition effectively rejects uninformative high–spatial frequency noise (fig. S5). We also observed that ZPs are demonstrably more efficient than other methods (*36*–*38*) in reconstructing the ground truth (fig. S6) and improving clustering performance (figs. S7 and S8) with different types and amounts of noise. Last, rotational symmetries within image patches are self-evident in the Zernike representation, which can be selectively turned on or off to determine relationships between class averages (compare Fig. 1, D and F, against Fig. 1, E and G). Hence, Zernike features are robust against rotational uncertainty. Our time and space complexity analysis (fig. S9) also shows that computing Zernike moments via matrix approximation (fig. S10) is about 7.8 times faster and 1.5 times more memory efficient than principal components analysis (PCA) (scikit-learn implementation).

Classifying noisy unseen motifs together based on similarities between their dominant Zernike features creates average motifs with even less noise, making downstream labeling more robust. Automatically creating such class averages is critical in the preprocessing of very noisy cryo–electron micrographs (*39*) and x-ray diffraction patterns (*40*). To do this classification flexibly, we introduce a FR clustering scheme, which generalizes the efficient uniform manifold approximation and projection (UMAP) scheme as a multistage force-based clustering algorithm (fig. S11 and movie S1). Following the comparison workflow in fig. S12, our FR scheme shows comparable performance with t-distributed stochastic neighbor embedding (*41*) and UMAP (*42*) with balanced datasets (fig. S13) and outperforms these two with imbalanced datasets (fig. S14). The resultant FR layout (Fig. 1, F and G) can be flexibly adapted to datasets with either discrete motif classes or manifolds of motifs by tuning the relative strengths and schedules of these forces (see Materials and Methods).

Our image patches typically enclose the neighboring atomic columns. This is an efficient way of extracting single-atomic column defects without supervision, which readily appear at the FR layouts (Fig. 1, F and G). These motif labels that are automatically derived from this classification, plus the relative spatial locations between pairs of motifs, can be hierarchically composed to discover simple relationships between structural motifs (Fig. 1H).

A hierarchy of the structural motifs found in Fig. 1 can be automatically constructed with the steps described in Fig. 2. This construction is most readily done when these motifs fall within a lattice. We perform a second round of unsupervised classification where cells on this lattice are classified by their motif compositions (Fig. 2B). The resultant motif-cells, which deviate from the perfect crystal, are, in turn, programmatically connected to similar adjacent motif-cells (Fig. 2, B and C). A hierarchy can be automatically formed from these connected (but spatially isolated) motif-cells, where the relationships between and occurrence rates of these motif-cells become apparent (Fig. 2D). Details are discussed in Materials and Methods.

The analyses above only require several seconds on a modest desktop computer (table S2). Hence, it can be easily adapted to give live feedback at electron microscopes.

**Fig. 3.**
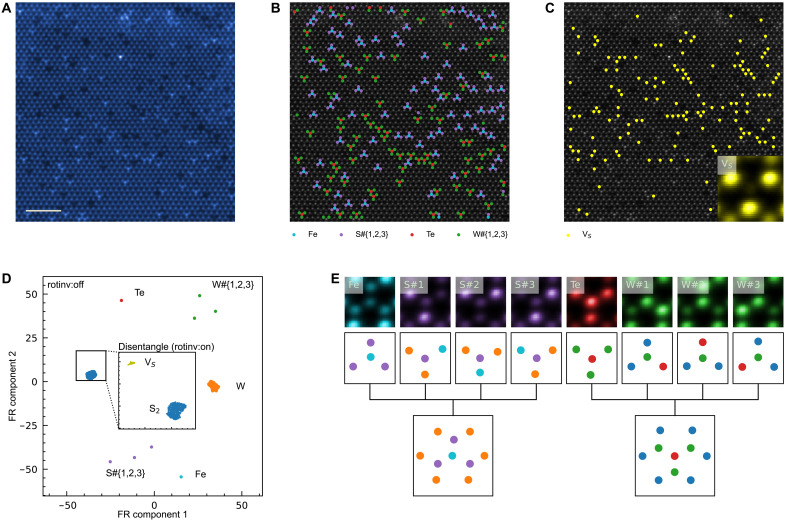
Comprehensive identification of dopant sites in quaternary alloy based on monolayer WS_2_. (**A**) Low-angle ADF-STEM image of monolayer WS_2_ doped with Fe and Te. Scale bar, 2 nm. Our framework readily identified (**B**) Fe-centered (turquoise) and Te-centered (red) motifs, as well as (**C**) single S vacancies V_S_ (yellow) within the WS_2_ lattice (gray). (**D**) FR clustering of Zernike features (rotation invariance turned off) shows the W-centered motifs (orange) and S-centered motifs (blue), plus the structural motifs that contain dopants (Fe and Te) or have peripheral S or W atom columns (i.e., S#{1,2,3} and W#{1,2,3}). Among the motifs comprising only W + S, those that are centered on S vacancies can be identified by turning on rotational invariance (inset). (**E**) For the motifs containing dopants, their spatial context can be summarized as a tree-like hierarchy with other motifs.

This framework readily generalizes to crystalline samples with higher defect densities (Fig. 3). An example of such is a monolayer WS_2_ doped with Fe and Te atoms (Fig. 3A), where the FR clustering of the Zernike features rapidly identifies more motif classes (Fig. 3D). Using only the spatial relationships between these motifs, more complex motifs can be hierarchically composed (Fig. 3E): Fe-centered defects that are surrounded by three S columns, which are, in turn, fenced in by W columns; Te-centered motifs that are surrounded by three W columns, which are, in turn, fenced in by S-centered columns. The original ADF-STEM micrograph can be readily reconstructed with these motifs (Fig. 3, B and C), where motifs of the background crystalline WS_2_ monolayer are hidden for clarity. A hierarchy of motif-cells, similar to but more complicated than that in Fig. 2, can be reconstructed for this sample (see fig. S15).

**Fig. 4.**
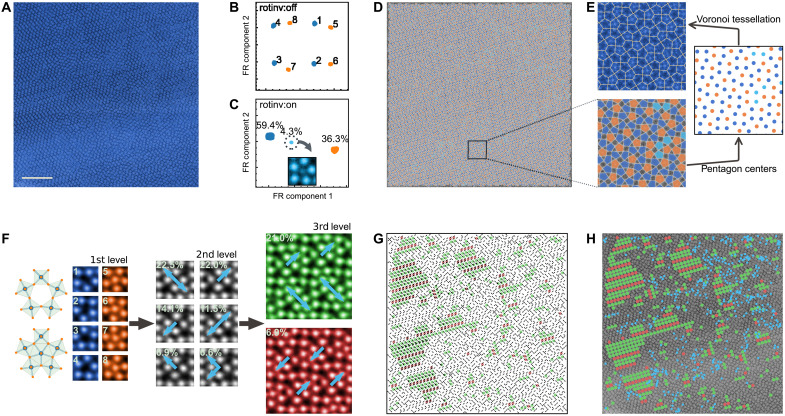
Hierarchy of motifs in POM Mo─V─Te─Nb─oxide shows previously unidentified but frustrated phase. (**A**) ADF-STEM image of the complex metal oxide with no apparent long-range order. Scale bar, 10 nm. (**B** and **C**) FR clustering of their Zernike features shows motifs that are centered on empty atom columns (dark blue), partially filled columns (light blue), or filled pentagonal atom columns (orange). (**D**) While the ADF-STEM image can be reconstructed with these three motifs in a quasi-random way, (**E**) the Voronoi tessellation of their centers forms a monohedral pentagonal tiling (type 4). (**F**) Hundreds of larger motifs can be composed from these filled/hollow pentagonal motifs in a three-level hierarchy (only significant nodes in hierarchy shown here). The most abundant motifs at the third level (green and red) are centered on four-column squares, labeled by arrows indicating the direction of filled pentagons from the centers. (**G**) Reconstruction of the ADF-STEM image with these arrows and (**H**) the largest dominant motifs clearly shows a previously unidentified phase that could tessellate the plane but is frustrated by other competing structural motifs.

Our framework can create powerful annotations for understanding structures that are too disordered for manual classification. An example of this is the large family of POMs, where multiple polyhedral transition metal oxyanion units link together to form complex 3D structures that hold promise for novel catalytic (*18*) and biomedical applications (*19*). Figure 4 (A to C) illustrates how our framework rapidly identified three types of pentagonal motifs in the Mo─V─Te─Nb─oxide POM and their relative abundances. These motifs resemble five MO_8_(M = Mo and Nb) octahedra arranged in a pentagon but with the following differences: 59.4% of these pentagons are empty; 36.3% likely surround either niobium (*Z* = 41) or molybdenum (*Z* = 42) atomic columns; 4.3% possibly surround vanadium (*Z* = 23) atomic columns (fig. S16).

These three fundamental pentagonal motifs tessellate the plane resembling a type 4 monohedral pentagonal structure (Fig. 4, D and E) (*43*). This tessellation, which canonically involves only a single species of irregular pentagon, is instead supported here by a spectrum of subtly distorted pentagons (fig. S17).

Hierarchically composing higher-level motifs from the two major types of single-pentagon motifs (Fig. 4F and fig. S18) reveals how these pentagonal units might have spontaneously assembled. Level 2 motifs comprise four pentagons that occupy the corners of a larger square; here, the putative vanadium-centered pentagons (light blue motifs in Fig. 4C) are omitted. The blue arrows directed toward the corners of these level 2 motifs indicate whether the pentagons at the specified corners are filled. In a completely disordered sample, one would expect 2^4^ = 16 such level 2 motifs that occur with equal probability. However, Fig. 4F shows a lower–configurational information entropy structure where >83% of these level 2 motifs are dominated by only six of these motifs. This low-entropy signature suggests the preferential attachment of pentagonal, but distorted, units during their solution-phase growth (*18*).

Even larger level 3 square motifs can be formed where each corner is now occupied by a level 2 square motif. Again, in a completely random and disordered structure, there should be 2^16^ = 65,536 level 3 motifs of equal probability. In addition, yet again, nearly a quarter of the field of view (Fig. 4G and fig. S19) is dominated by only two distinct level 3 motifs that conspire to form a previously unreported ordered structure with an approximate 3:1 ratio. From Fig. 4G, it is clear that the growth of this previously unidentified ordered structure was entropically frustrated by a large family of competing motifs rich in Mo and Nb columns (fig. S20). This frustration could have been further abetted by the putatively V-enriched motifs, which appear in the regions between these ordered structures as indicated in turquoise dots (Fig. 4H).

**Fig. 5.**
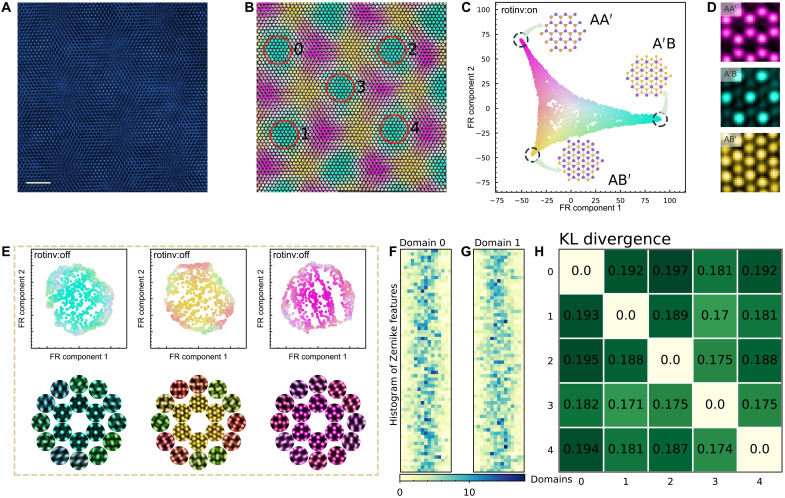
Reconstructing variations between domains in twisted bilayer MoS_2_. (**A**) Low-angle ADF-STEM image of bilayer MoS_2_ with 3.15° interlayer rotation. Scale bar, 2 nm. (**B**) Zernike features in this image, colorized by our framework, show the coexistence of AA′, AB′, and A′B type domains. (**C**) FR layout of these Zernike features, with the rotational invariant mode turned on. This layout is anchored at its three vertices by the corresponding atomic models from the AA′, AB′, and A′B phases, whose average experimental motifs are shown in (**D**). All the features in (C) are colored on the basis of their distances from these three anchor motifs. (**E**) Top row shows the FR layout of Zernike features from A′B, AB′, and AA′ domains (left to right) with the rotational invariant mode off. Image patches averaged from the corresponding features in the bottom row show how these features spatially fill the A′B, AB′, and AA′ domains. (**F**) Binned histograms of all Zernike features in A′B domain 0 in (B) sequentially projected onto its 66 feature dimensions (each shown as a separate row); (**G**) identically binned histogram from features in A′B domain 1 of (B). (**H**) Pairwise KL divergences between the Zernike feature histograms from the five A′B domains circled in (B).

When the variations in the sample are more continuous than combinatorial, constructing motifs can be uninformative. Consequently, building hierarchies from such motifs are potentially misleading. A good example of such samples is the family of van der Waals heterostructures whose electronic and structural properties can be manipulated by introducing relative rotations between atomically thin ordered layers. Figure 5 shows an ADF-STEM micrograph of two hexagonal MoS_2_ layers that were mutually rotated by approximately 3.15° (fig. S21), creating a characteristic Moiré pattern (Fig. 5A). Although our Zernike-based framework automatically identifies a finite number of high-symmetry structures from the micrograph (Fig. 5B), the continuum of possible relative rotations and shifts between layers causes the experimentally observable variations between these features to be more continuous than discrete in nature. Hence, unlike the FR layouts in Figs. 1, 3, and 4, the FR clustering map of these Zernike features, which were made rotationally invariant, cannot be meaningfully classified as a finite number of motifs (Fig. 5C).

Despite the continuous variations between the features in this bilayer MoS_2_ sample, anchor motifs can still be induced among them. These anchor motifs, corresponding to the recognizable AA′, A′B, and AB′ idealized domains of this twisted bilayer, are still evident in FR layout in Fig. 5 (C and D). Unlike the motifs in Figs. 1, 3, and 4, which were created from the averages of abundant pseudo-copies of similar features in the sample, the anchor motifs in the twisted bilayer case represent a much smaller fraction of all possible features. Nevertheless, all the features in the micrograph can be quantitatively measured and labeled from these anchor motifs (Fig. 5B). If we separately classify the features closest to these anchor motifs, a hierarchy of motifs emerges describing how AA′-centered, A′B-centered, and AB′-centered domains can be spatially composed (Fig. 5E).

Experimentally realized twisted bilayers will contain imperfections. These imperfections, through the variations between domains or perturbation (*44*), can be readily quantified in our framework. Figure 5 (F and G) shows the projected histograms of Zernike features from similar regions centered on two A′B motifs 0 and 1 of Fig. 5B. While these two sets of histograms are broadly similar, their visible variations betray differences between these two presumptive A′B domains. Pairwise comparisons of the Kullback-Leibler (KL) divergence between these Zernike feature histograms for different A′B domains (Fig. 5H) show divergences (0.184 ± 0.025) that are larger than expected from known nuisance parameters (fig. S22). Simply put, despite idealized descriptions of these domains (*45*), they contain quantifiably different groups of features.

## DISCUSSION

Deeply rooted in our framework is the critical notion of structural motifs. Motifs coarse-grain and hence simplify the staggeringly large space of possible atomic configurations in our sample. Each motif class can accommodate variations in features due to uninformative nuisance parameters (e.g., noise, scanning errors, detector noise, etc.) or geometric parameters (e.g., small strain fields). With sufficiently high-resolution images, some of these parameters can be unambiguously identified and coarse-grained away. Thereafter, insights about a sample’s state and dynamics can be distilled more readily.

This coarse graining is intimately linked to the fact that interpreting a motif class is more robust against noise and aberrations compared to single noisy image patches. When the signal is sufficiently larger than the noise, such coarse graining reveals structural motifs (e.g., Fig. 3) despite local strain fields (see POM structure distortion map; fig. S17). Admittedly, however, for very noisy measurements, it will be impossible to discern structural features in the motifs from noise. In addition, the ZP representation offers robustness against the types of scanning drift that were encountered in the experimental images presented here. However, again, this robustness will be compromised when this scanning drift becomes sufficiently severe.

These motifs are the essential building blocks for organizing the structural complexity of samples into interpretable hierarchies. In a perfectly periodic structure, the number of atom-centered motifs at any level of this hierarchy equals the number of unique and visible atomic columns within the unit cell (fig. S1). However, the multiplicity of these motifs will increase with point defects (fig. S23) and interdomain boundaries (figs. S24 and S25). Further introducing random disorder to this periodic structure (e.g., from vacancies, interstitials, translations, and distortions) causes the number of complex motifs to rapidly rise as we build toward more complex hierarchies in our Mo─V─Te─Nb─oxide sample (figs. S20 and S26). The rate of this rise is a measure of a sample’s configurational entropy and loss of long-range order. This entropy also specifies how higher-level motifs can be efficiently and combinatorially represented with lower-level fundamental motifs (Fig. 2 and figs. S15 and S27).

The dominant hierarchy of motifs in Fig. 3F is strongly influenced by the dominant self-assembly pathways in the sample. In contrast, the sample also reveals a much larger gamut of thermodynamically accessible but less likely higher motifs (figs. S20 and S26). The exercise of constructing these hierarchies may reveal crucial clues about preferential attachment and free-energy barriers during multistep nucleation and growth of ordered phases (*46*). In addition, these motif hierarchies also provide statistically grounded structural waypoints to guide (or check) ab initio calculations of viable structures, dynamics, and downstream function (*2*, *47*). These ideas can be readily extended to other atomic resolution structures, such as those collected using scanning tunneling microscopes (fig. S28).

To conclude, we have described a framework based on Zernike features and FR clustering to extract and represent motifs from complex atomic resolution micrographs and accelerate downstream labeling. This framework continues to exploit the spatial context between simple motifs to learn a hierarchical composition of higher-level motifs that can reconstruct an image from the bottom up. By explicitly inducing this motif hierarchy in a sample, we can quantify and interpret the degree and types of complexity in a sample. Ultimately, these motifs help us coarse-grain disorder and/or complexity in materials to a degree where new knowledge readily emerges and, in turn, inspires insightful hypotheses and theoretical studies. The capability to algorithmically construct structure hierarchy via microscopy images has the potential to alter how materials scientists characterize and interpret complex materials structures, usually not readily evident to human eyes, in areas such as oxide thin films (*48*), metal halide perovskite (*49*), and colloidal crystal (*50*). Hence, our techniques offer a novel and rapid approach to extracting multilevel structural information from atomic-level microscopy images and establishing statistically multiscale structure-property links, paving the way to rapid and automatic discovery of next-generation nanomaterials with complex and unknown features.

## MATERIALS AND METHODS

### Growth of WS_2_ thin films with Te and Fe dopants

Tellurium (Te) powder was placed into a quartz boat at a temperature of *T*(Te) ≈ 450°C. WO_3_ and FeS_2_ powders were put in a ceramic boat inside the quartz tube at the center of heating zone. A Si/SiO_2_ substrate with a clean surface was put on the boat. The growth temperature was set at about 800°C, and the growth time was 30 min. The flow rate of the argon (Ar) carrier gas was 90 standard cubic centimeters per minute.

### Growth of MoSe_2_ thin films via molecular-beam epitaxy

SiO_2_ substrates were degassed in the same chamber for 1 hour and annealed at 500°C for 10 min. Mo and Se powders were evaporated from an electron beam evaporator and a Kundsen cell, respectively. During growth, the temperature of the SiO_2_ substrates was maintained at 500°C, with a flux ratio between Mo and Se of ∼1:10, and chamber pressure was kept at ∼9 × 10^–10^ torr. Monolayer and bilayer MoSe_2_ can be obtained when the growth temperature is set at 250°C.

### STEM sample preparation

As-grown transition metal dichalcogenide (TMDC) films were first identified by optical microscopy. Cu QUANTIFOIL TEM grids were placed onto the target region of TMDC thin films followed by an isopropyl alcohol–assisted polymer-free lift-off method. The TEM grids were annealed in an ultrahigh vacuum chamber (~1 × 10^–9^ torr) at 180°C for 10 hours before STEM imaging to eliminate surface contamination.

### STEM characterization

STEM-ADF imaging was carried out on an aberration-corrected JEOL ARM-200F equipped with a cold field-emission gun at 80 kV, if otherwise stated. Two sets of detector acceptance angle were adopted. A higher detector range (68 to 280 mrad) was used for MoSe_2_ characterization. A lower detector angle range (30 to 68 mrad) was adopted for WS_2_ imaging for improved contrast of S vacancy sites. A dwell time of 19 μs/pixel was set for scanning imaging mode. HAADF-STEM imaging of the POM structure was performed on UltraSTEM 200 (operated at 200 kV).

### Synthetic data generation

Synthetic datasets here are used to evaluate popular dimension reduction methods (PCA, t-SNE, and UMAP) against the FR clustering. We have listed the details of synthetic data in table S3. Simple synthetic motifs in figs. S1 to S5 are directly calculated by adding different 2D Gaussian functions. In addition to binary classes of motifs with different *n*-fold symmetry, synthetic bilayer patterns used to evaluate KL divergence (fig. S22) are generated by convolution with corresponding Gaussian kernels in the Fourier space using sufficient up-sampled grids. Then, the generated Moiré patterns are then rescaled to adapt to the resolution of experimental data.

### Identification of feature points

Identification of feature points follows three steps: smoothing, maximum filtering, and locating the points. The workflow is detailed in fig. S29. The key to successfully extracting feature points is to obtain smooth versions of raw images (step 1). Depending on image quality and image conditions, different smoothing schemes are adopted. For images with high signal-to-noise ratio (SNR), Fourier space filtering was implemented to keep 10% of the lowest-frequency components. For images with low SNR, singular value decomposition (SVD)–based method (*51*) was applied to the image. We adopted the SVD-based method for images in this work unless stated otherwise. The smooth image was dilated by a local maximum filter (step 2). The feature points are the locations where input image is equivalent to the dilated version (step 3). In some cases, this identification scheme conservatively overcounts the number of feature points, and feature points can be further reduced via symmetry response of Zernike features or selected in FR clustering scheme. It is noted that our ZP representation is more robust to position perturbation compared with PCA (fig. S30). Although ZPs demonstrate near-perfect tolerance within 4 pixels, we would recommend improving the accuracy of feature point identification in extreme cases to achieve an overall better performance in downstream analysis.

### Determination of motif patch size

Patch sizes have to be sufficiently large to capture meaningful local symmetries that are persistent in the sample. These symmetries would be efficiently and interpretably encoded in the Zernike representation of these image patches. Empirically, we found this encoding satisfactory when the side length of the image patches matches the average length of the repeating unit. The latter can be automatically detected using the radial distribution function (see workflow in fig. S31). Patch sizes smaller than this recommendation tend to offload local symmetry information to the hierarchies that their consequent motifs form, which may not be as readily interpretable.

### Dimensional reduction with PCA

Let the initial set of feature vectors extracted from the raw image be denoted *X* = {*x*_1_, *x*_2_, ⋯, *x_i_*, ⋯, *x_n_*∣*x_i_* ⊆ *R^m^*}, where *n* labels the number of features (image patches), and *m* is the length of feature vector that stores Zernike moments. We then use linear PCA to identify the main components of covariance between these features. Thereafter, we reduce the features’ dimensionality by projecting them into the PCA components that capture the largest feature-feature variations. Here, we project all Zernike features (*j* ≤ 65, *m* = 66) obtained from STEM images into the two largest PCA components (*X* ↦ *Y* = {*y*_1_, *y*_2_, ⋯, *y_i_*, ⋯, *y_n_*∣*y_i_* ⊆ *R*^*d* = 2^}, where *d* labels PCA components).

### FR clustering

Our two-stage relaxed clustering comprises a repulsion-dominated stage, followed by an attraction-dominated stage. The repulsion-dominated stage allows adequate separation distance between structural motifs whose features are mutually most dissimilar. The attraction-dominated stage then adjusts the strength of attraction force to make sure that each motif cluster is compact and that clear decision boundaries can be drawn between clusters. The forces used here and others (*41*, *42*, *52*) are described in table S4 and elaborated in Supplementary Text. The default hyperparameters in the attraction-dominated and repulsion-dominated stages are {α = 1, β = 1, *n* = 0, *m* = 2} and {α = 5, β = 1, *n* = 2, *m* = 5}, respectively. Although all examples shown in this work use this default set of hyperparameters, users can optimize them for their own imaging needs.

If a feature (e.g., in Fig. 1C) is another feature’s *k*-nearest neighbor (determined once from the feature matrix *X*), then attractive forces will pull them together in this PCA-reduced space; otherwise, randomly chosen *k*′ non-neighbor pairs are mutually repelled via a stochastic scheme. Exemplary results are shown in Fig. 1 (F and G), where *k* = 10 and *k*^′^ = 5. These forces iteratively move feature pairs (*Y*) in the PCA-reduced layout and are inversely proportional to their mutual separation on this plane and weighted by a similarity metric between the feature pairs in their original Zernike space (*X*). To provide additional control, we alter the relative strength of these attractive and repulsive forces into a repulsion-dominated stage, followed by an attraction-dominated stage. For numerical stability, these forces are gradually relaxed from a maximum in both stages. The hyperparameters involved are illustrated in fig. S11.

The evolution of the features that resulted in Fig. 1 (F and G) reveals how the two-stage relaxed clustering creates decision boundaries between structural motifs. Features suffer a rapid global dilation during the initial repulsion-dominated stage. During this stage, approximate class boundaries quickly emerge while keeping their *k*-similar features nearby. This dilation is slowed by a force-relaxation schedule. Subsequently, during the attraction-dominated stage, similar features coalesce into well-separated centroids without a global contraction of all the features. This ordering of the stages is crucial for creating clear decision boundaries between features, which can be optimized with the hyperparameters in table S3 and Supplementary Text. Further comparison is further discussed in figs. S13 and S14.

### Automatic construction of motif-cell hierarchy

Here, we describe the procedure used to programmatically produce the motif-cell hierarchies in Fig. 2 and fig. S15. A variant of this approach is used for the POM dataset (figs. S26 and S27).

1) Use the feature points on an image to define cells that tessellate the entire image. This can be done in two ways: automatically locating the lattice vectors in a quasi-periodic sample, which gives a regular grid of cells; or a Voronoi construction where the resultant cells might not be periodic.

2) Each of the lowest-level structure motifs labeled by FR clustering is associated with its encompassing cell. This produces motif-cells similar to those seen in Fig. 2B.

3) Single-cell motif-cells (e.g., rhombuses in Fig. 2B) are rapidly classified by the set membership of their motifs. These unique single-cell motif-cells form the lowest level of the hierarchy that we will build below. In a mostly crystalline sample, the most frequently observed single-cell motif-cells will belong to the periodic unit cells of the crystal. Here, we ignore these dominant motif-cells to focus on how nondominant motif-cells self-assemble.

4) If the cells’ positions are described by lattice vectors $i\vec{u}+j\vec{v}$, then each cell’s position can be uniquely represented using only its lattice index [i.e., (*i*, *j*)].

5) Each nondominant motif-cell is “grown” by automatically connecting it to adjacent motif-cells in their von Neumann neighborhood. A higher-level motif-cell is created when all the neighbors of its constituent motif-cells have all been connected onto this larger motif-cell.

6) These larger, higher-level connected motif-cells are ordered by levels according to the number of cells that each contains (Fig. 2D). Higher-level motif-cells with the same number of cells are compared to remove duplicates up to an overall 90° rotation.

7) To build a hierarchy from these motif-cells, we exhaustively check whether each lower-level (i.e., smaller) motif-cell is a subset of any of the higher-level motif-cells (accounting for all possible translations but not rotations). For visual clarity in the hierarchies of Fig. 2D and fig. S15, edges are only drawn between higher-level motif-cells and their subsetted lower-level motif-cells should the difference in their levels be smaller than some integer threshold.
