# Supplementary material for: Learning motifs and their hierarchies in atomic resolution microscopy
Source: Sci Adv. 2022 Apr 13;8(15):eabk1005. doi: 10.1126/sciadv.abk1005 (PMC9007509; doi:10.1126/sciadv.abk1005)
Supplement: Supplementary file 1 — Supplementary Text Figs. S1 to S31 Tables S1 to S4 [file sciadv.abk1005_sm.pdf]

Science Advances
AAAS

Supplementary Materials for

Learning motifs and their hierarchies in atomic resolution microscopy

Jiadong Dan, Xiaoxu Zhao, Shoucong Ning, Jiong Lu, Kian Ping Loh, Qian He, N. Duane Loh*, Stephen J. Pennycook*

*Corresponding author. Email: duaneloh@nus.edu.sg (N.D.L.); stephen.pennycook@cantab.net (S.J.P.)

Published 13 April 2022, *Sci. Adv.* **8**, eabk1005 (2022)
DOI: 10.1126/sciadv.abk1005

The PDF file includes:

Supplementary Text
Figs. S1 to S31
Tables S1 to S4
Legend for movie S1

Other Supplementary Material for this manuscript includes the following:

Movie S1

Supplementary Text

Finite Number of Motifs in Crystal sample

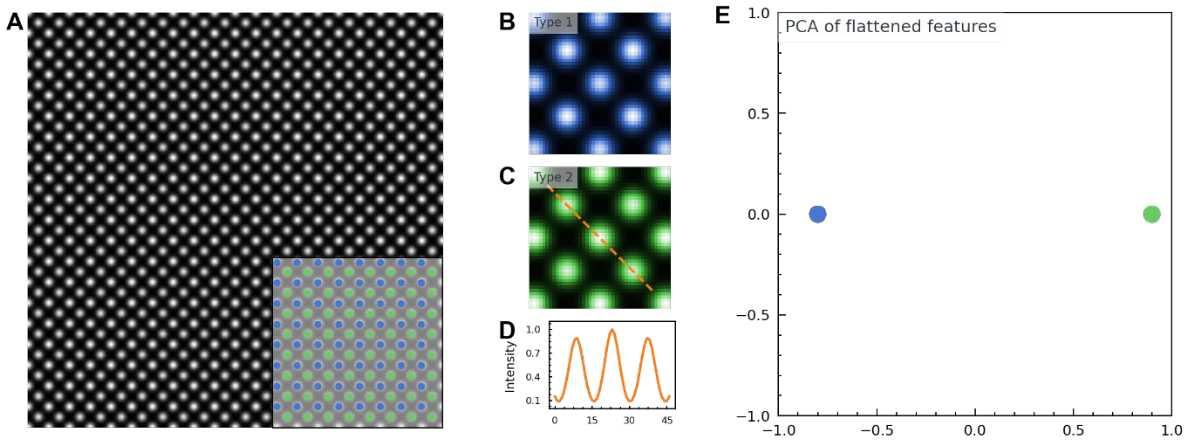

Fig. S1 Finite number of motifs in a synthetic crystal sample. (A) Synthetic image of a crystal sample with plane group $p4m$, which comprises two types of motifs: a motif centered with a dimmer blob and a motif centered with a brighter blob as shown in (B) and (C) respectively. (D) Line profile showing the intensity difference in (C). (E) The PCA projection of flattened features demonstrates the discrete nature of this sample.

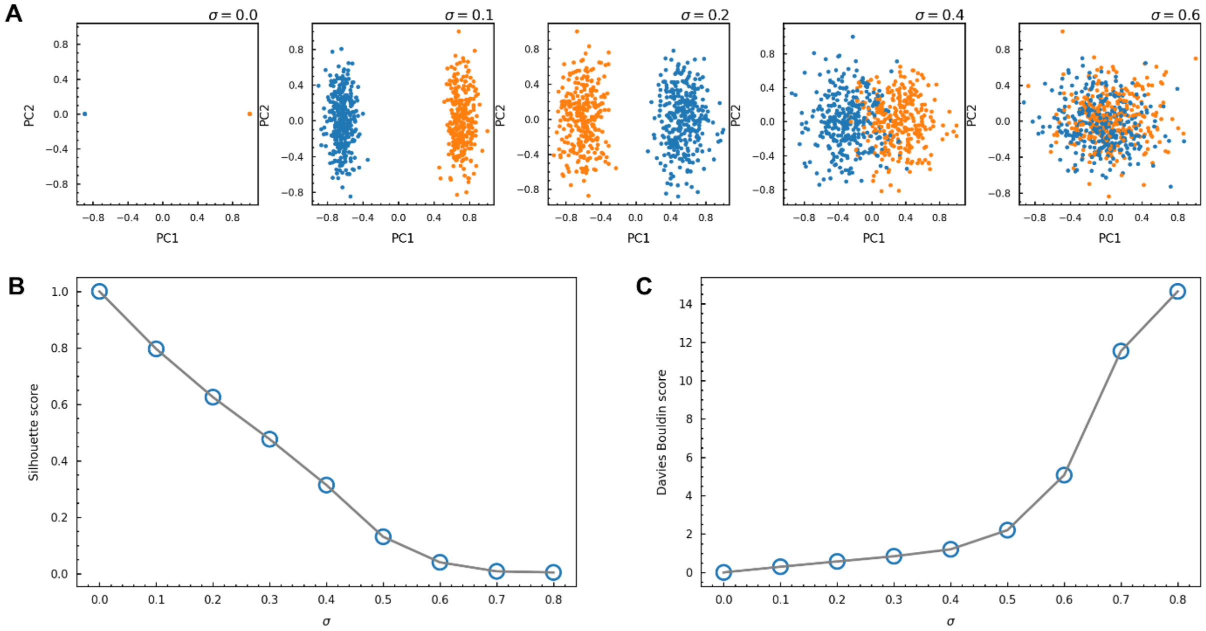

Fig. S2 Cluster broadening due to addition of Gaussian noise with standard deviation σ. (A) The PCA projections of flattened features from the synthetic crystal in Fig. S2 with various Gaussian noise levels. With an increase of σ values, two clusters representing two types of motifs

will finally overlap. To evaluate the degree of overlap, the Silhouette score (B) and Davies-Bouldin score (C) are plotted against different σ values.

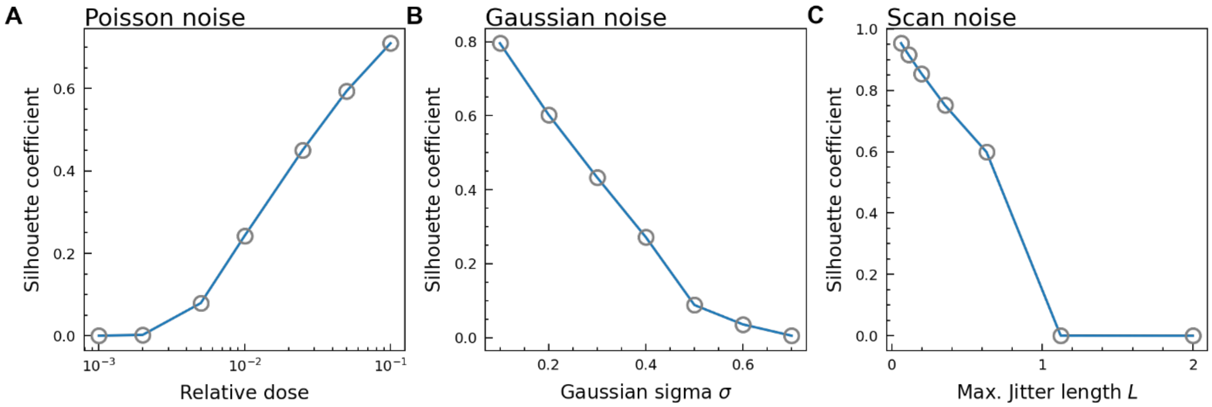

Fig. S3. Cluster broadening in three different noise settings: Poisson noises, Gaussian noises and scan noises. Synthetic dataset parameters: $\{n1 = 5000, n2 = 5000, s = 128, \sigma = 7, l = 32, A = 0.8\}$.

Conveniently, STEM images of crystalline samples contain only a finite number of atom-centered motifs, which often correspond to the discrete nature of the low dimensional representation of these motifs. This discrete property is closely related to the fact that crystal structure can be concisely described by a unit cell that only contains a finite number of atoms. We used synthetic data composed of an array of Gaussian blobs to validate this statement. Fig. S1A displays a synthetic image of a crystal sample with plane group $p4m$, which contains two types of motifs as indicated in the inset image and Fig. S1 (B and C). Fig. S1 D shows the intensity difference of two types of blobs. The maximum intensity of the central blob in type 1 and 2 motifs is 0.8 and 1. Fig. S1 E is the PCA projection of flattened features demonstrating the discrete nature of this sample.

Fig. S2 illustrates the broadening of clusters from synthetic data in Fig. S1A with increasing Gaussian noise ($0 \leq \sigma \leq 0.8$). Fig. S2A selectively shows the PCA layouts in different σ values. Fig. S2B and Fig. S2C depict the Silhouette score and David-Bouldin score against σ. The decreasing trend in the Silhouette scores, and the increasing trend in the David-Bouldin scores quantitatively demonstrate that these two types of atom-centered motifs are increasingly difficult to differentiate with increasing noise.

Fig. S3 shows the cluster broadening effect is also evident in Poisson noises and scan noises settings. Here we used a synthetic binary class dataset from Table. S3.

<u>Zernike Polynomials and Zernike moments</u>

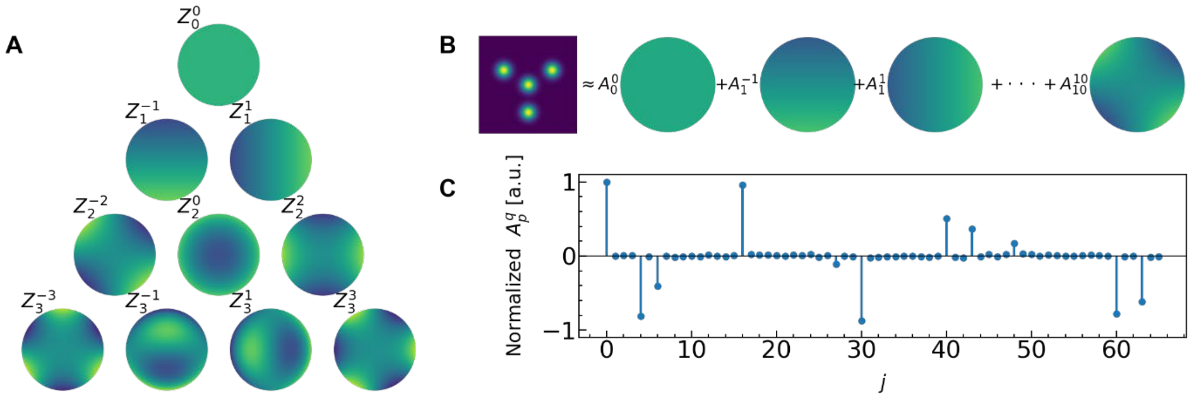

Fig. S4 Zernike polynomials and Zernike moments. (A) The first 10 Zernike polynomials arranged in a pyramid form. Each polynomial term is labelled by Z_p^q, where p is the radial index and q is the azimuthal index. All Zernike polynomials are vertically arranged by p and horizontally ordered by q. (B) The decomposition of image patch to a linear combination of Zernike polynomials. (C) The coefficients for each polynomial can be grouped to form a compact representation of the original patch.

The ZPs are a complete set of orthogonal basis functions defined in the unit disk denoted by the double indexing scheme $Z_p^q(\rho,\theta)$ (Fig. S4), where p is a nonnegative integer, and $q = \{-p, -p+2, -p+4, \dots, p\}$ for a given p. The double indices (p, q) are ordered into a single index $j = (p(p+2)+q)/2$. Each ZP consists of a normalization term N_p^q, a radial term $R_p^{|q|}$, and an azimuthal term $sin(q\theta)$ or $cos(q\theta)$:

$$Z_p^q(\rho,\theta) = \begin{cases} N_p^q R_p^{|q|}(\rho)\cos(q\theta); & for\ q \geq 0 \\ -N_p^q R_p^{|q|}(\rho)\sin(q\theta); & for\ q < 0 \end{cases}$$

Here, $R_p^{|q|}$ and N_p^q are given by

$$R_p^{|q|}(\rho) = \sum_{k=0}^{\frac{p-|q|}{2}} \frac{-1^k(p-k)!}{k!\left(\frac{q+|q|}{2}-k\right)!\left(\frac{p-|q|}{2}-k\right)!}\rho^{p-2k},$$

and

$$N_p^q = \sqrt{\frac{2(p+1)}{1+\delta_{q0}}},$$

where δ_{q0} is the Kronecker delta.

Fig. S4 shows a visual depiction of the first 10 Zernike polynomials. The order of the polynomial is determined by the indices p and q. In fact, the double indices (p, q) are ordered into a single index j, *i.e.*, Z_p^q is equivalent to Z_j. Table S1 shows the conversion between (p, q) to j, for which the mathematical relation is expressed below,

$$j = \frac{p(p+2)+q}{2}.$$

The azimuthal component of Z_p^q is either $sin\ (q\theta)$ or $cos(q\theta)$. In fact, it can be generalized to a complex form using Euler's formula $e^{ix} = cos\ (x) + isin(x)$. Hence, Zernike polynomials in complex-valued form can be expressed as

$$Z_p^q(\rho,\theta) = N_p^q R_p^q(\rho)e^{iq\theta}\ .$$

It is noteworthy that $Z_p^q(\rho,\theta)$ is complex valued if derived in complex form, which is useful to prove the rotation-invariance properties of Zernike moments in section Rotational Invariant form of Zernike Moments.

Any square-integrable functions $f(\rho,\theta)$ within a unit disk can be decomposed into an infinite series comprising weighted Zernike polynomials:

$$f(\rho,\theta) = \sum_{p=0}^{\infty} \sum_{q=-p}^{p} A_p^q Z_p^q(\rho,\theta), \quad p - |q| = even$$

where the coefficients A_p^q is can be calculated as

$$A_p^q = \int_0^{2\pi} \int_0^1 f(\rho,\theta) Z_p^q(\rho,\theta)\rho d\rho d\theta\ .$$

Rotational Invariant form of Zernike Moments

The image function defined in the unit disk $D = \{(\rho, \theta): 1 \leq \rho \leq 1, 0 \leq \theta \leq 2\pi\}$ is denoted by $f(\rho, \theta)$. If a rotation through an angle α is operated on the image function $f(\rho, \theta)$, a rotated version of image function $f^R(\rho, \theta)$ is obtained, and it relates to the original image function by,

$$f^R(\rho, \theta) = f(\rho, \theta - \alpha)$$

The complex-valued Zernike moments of the original image function $f(\rho, \theta)$ is

$$A_p^q = \int_0^{2\pi} \int_0^0 f(\rho, \theta) Z_p^{q*}(\rho, \theta) \rho d\rho d\theta = \int_0^{2\pi} \int_0^0 f(\rho, \theta) R_p^q(\rho, \theta) e^{-jq\theta} \rho d\rho d\theta$$

The Zernike moments of the rotated image function $f^R(\rho, \theta)$ in the same polar coordinate is

$$A_{R_p}^q = \int_0^{2\pi} \int_0^0 f(\rho, \theta - \alpha) R_p^q(\rho, \theta) e^{-jq\theta} \rho d\rho d\theta$$

Let $\theta_1 = \theta - \alpha$,

$$A_{R_p}^q = \int_0^{2\pi} \int_0^0 f(\rho, \theta_1) R_p^q(\rho, \theta) e^{-jq(\theta_1 + \alpha)} \rho d\rho d\theta$$

$$= e^{-jq\alpha} \int_0^{2\pi} \int_0^0 f(\rho, \theta_1) R_p^q(\rho, \theta) e^{-jq(\theta_1)} \rho d\rho d\theta = A_p^q e^{-jq\alpha}$$

This shows that $A_{R_p}^q$ and A_p^q differ by a phase shift that is closely related to the rotational angle α. The phase difference of $A_{R_p}^q$ and A_p^q indicates that the magnitudes of complex-valued Zernike moments remain identical to those prior to the rotation, *i.e.*, $\left|A_{R_p}^q\right| = \left|A_p^q\right|$.

Noise rejection of Zernike representations

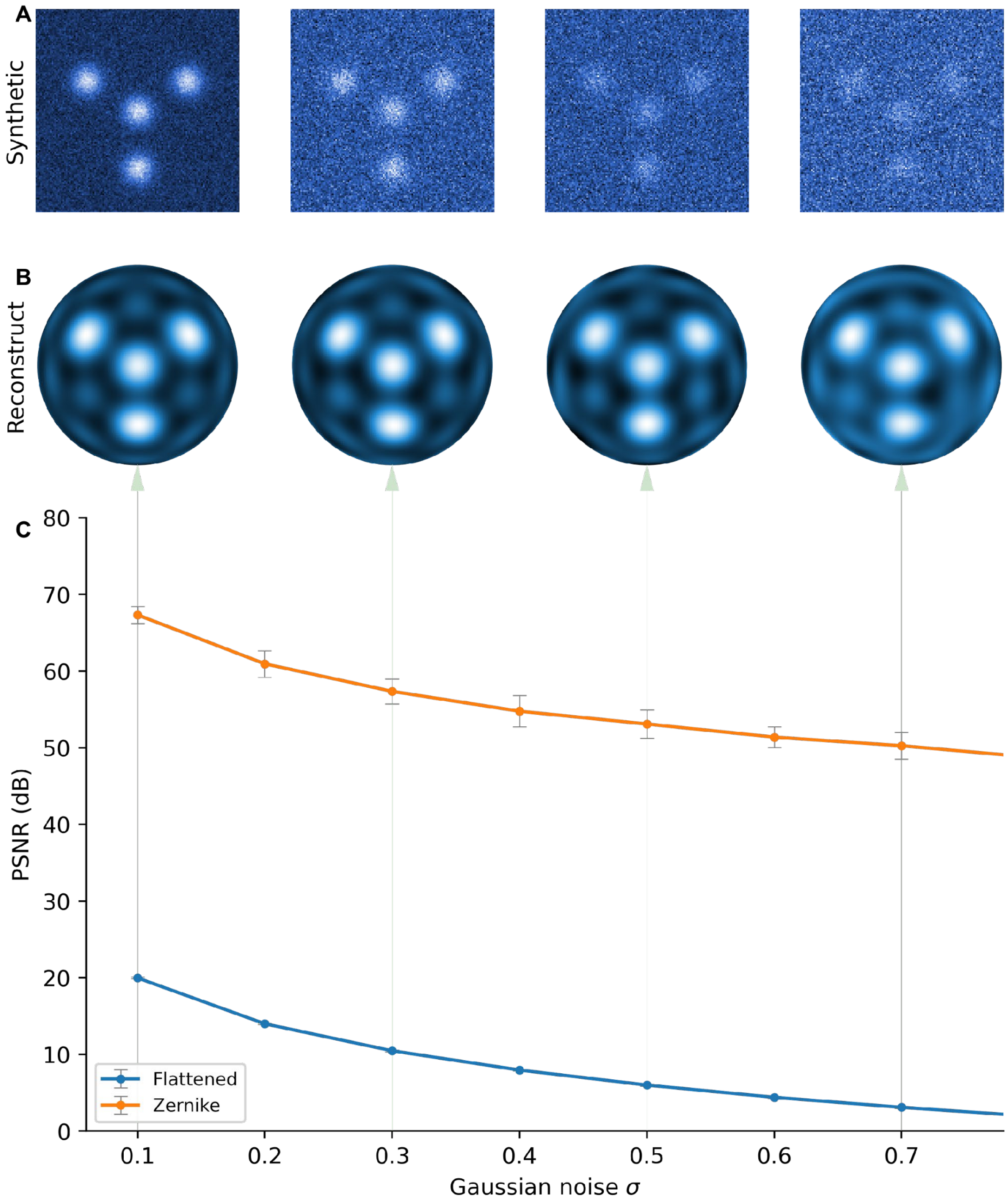

Fig. S5 Evaluate noise rejection using synthetic patches with 3-fold rotational symmetry. (A) Synthetic patches with increasing levels of Gaussian noise ($\sigma = 0.1, 0.3, 0.5, 0.7$). (B) The reconstructed patches from the first 66 terms ZPs indicate the main three-fold rotational symmetry even for high levels of noise. (C) Comparison of PSNR of two different feature representations: flattened image representation (blue) and Zernike representation (orange). The Zernike representation shows higher PSNR values in all levels of σ values.

The truncated ZP representations can effectively reduce noise. Much of the high spatial frequency measurement noise in high-resolution micrographs is predominantly contained in higher-order Zernike moments. Furthermore, the $p \leq 10$ Zernike projection of an image patch already captures a wide range of possible shapes and arrangements of atomic columns and defects. Therefore, by truncating ZPs beyond p=10 effectively allows us to reject higher spatial frequency measurement noise. As shown in Figure S5, with a very low value of PSNR (1.81), the reconstruction patch still shows the main three-fold symmetry which is consistent with the Zernike moments plot. Fig. S5 shows that the low spatial frequency features of noisy images are at least three orders of magnitude more detectable than the putative input peak signal to noise ratio in different symmetry configurations.

Comparison of ZPs and PCA from patch reconstruction

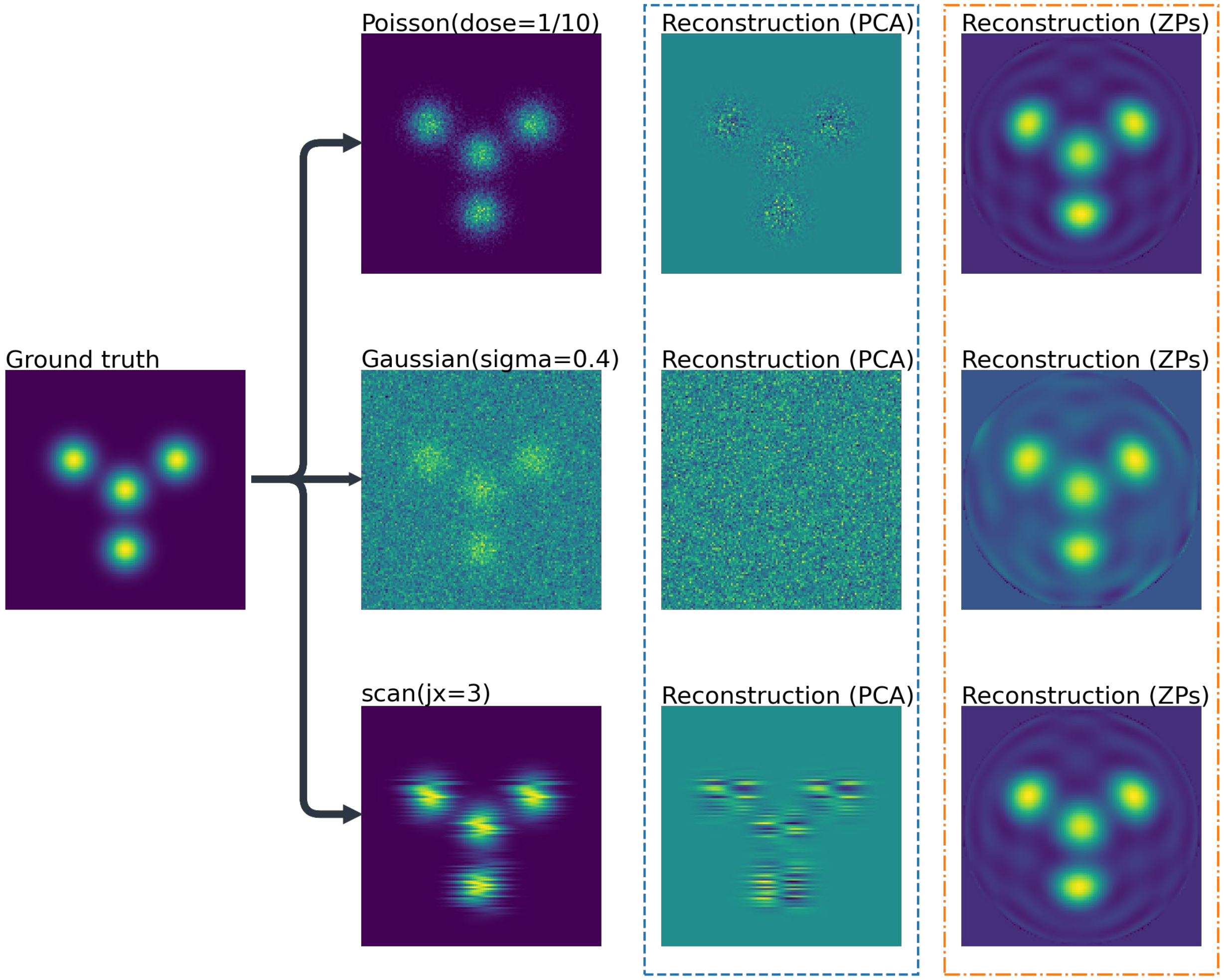

Fig. S6. Comparison of Reconstruction results using PCA and ZPs in different noise settings. ZPs reconstructions use the first 136 (corresponding to p=15 and q=15) terms of Zernike

polynomials. PCA reconstructions are computed from 500 stacks of synthetic patches, and truncated using the same number of components as ZPs.

To enrich the discussion towards why we choose ZPs and provide further evidence of the usefulness of our method, we have added more comparison analysis to validate our point. We have basically two paths to compare ZPs and PCA: compare the reconstruction results from PCA and ZPs both truncated to the same number of components; or compare clustering performance of features reduced using PCA or ZPs.

Fig. S6 shows the reconstruction results using PCA and ZPs when truncated to the same number of components in three different noise models. After applying only a moderate noise in all three scenarios, we observed that ZPs (orange rectangle) outperform PCA (blue rectangle) in recovering the spatial location and contrast of the ground truth patches. We have to point out that PCA is an effective image denoising method when implemented in overlapping patch-based singular value decompositions (SVD) method. Poisson noise is significantly reduced when overlapping patches are grouped as input into the SVD-related denoising algorithm. In representing features from patches using PCA, it contains variations from noise components if pre-pre-processing/denoising is lacking.

Comparison of ZPs and other dimension reduction methods via cluster performances

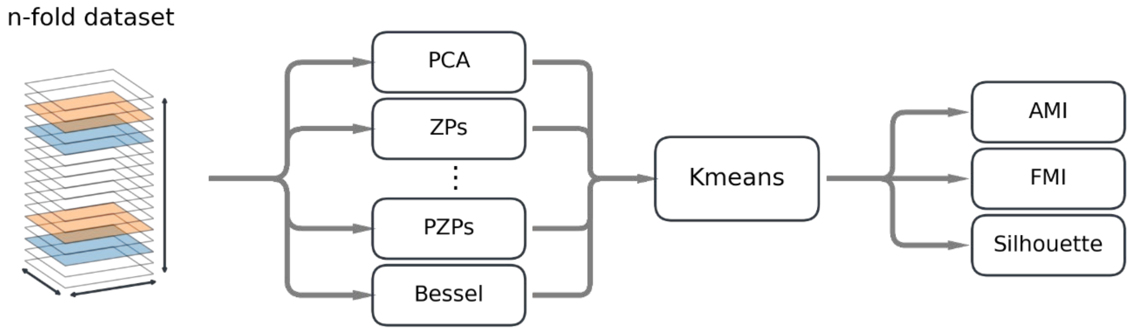

Fig. S7. Workflow to compare different representation (dimensional reduction) methods via clustering performance scores.

The advantages of ZPs over PCA are even clearer if evaluated based on clustering performance scores via a scheme shown in Fig. S7. The synthetic dataset (of different noise levels) is firstly represented via different dimension reduction techniques, then the reduced features are clustered using the KMeans algorithm. The predicted labels from Kmeans combined with ground truth labels are used to calculate different clustering performance scores including adjusted mutual information (AMI), Fowlkes–Mallows index (FMI) and Silhouette coefficient.

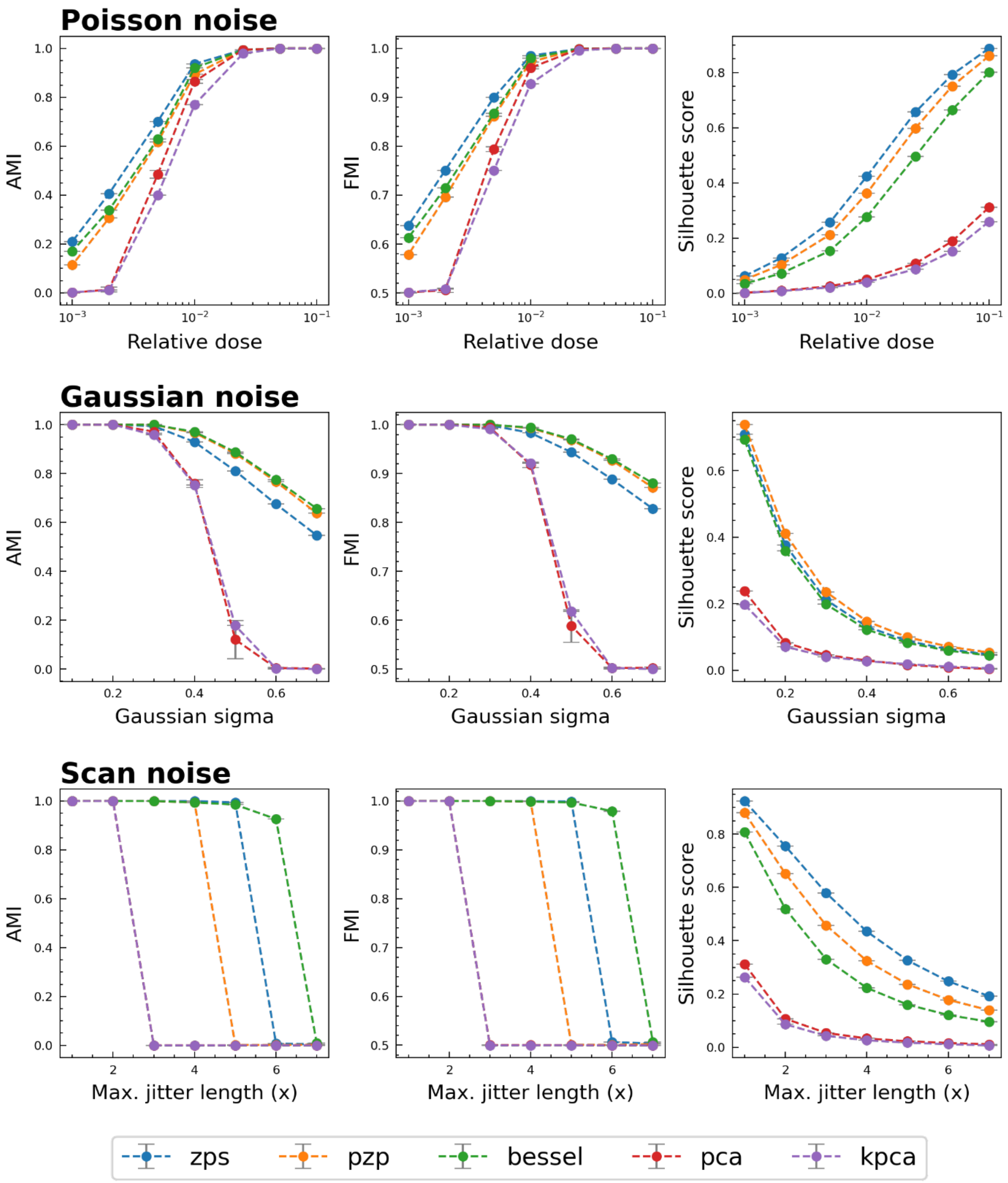

Fig. S8. Comparison of different representation (dimension reduction) methods. Top panels: AMI, FMI and Silhouette scores of different representation methods in *Poisson noise* setting. Middle panels: AMI, FMI and Silhouette scores of different representation methods in *Gaussian noise* setting. Bottom panels: AMI, FMI and Silhouette scores of different representation methods in *scan noise* setting.

Fig. S8 shows a complete comparison of these scores in different noise settings using various representation methods. In all three noise models, fixed-bases methods (*e.g.*, ZPs, PZPs and Bessel) outperform PCA and kernel PCA. In particular, ZPs show the best clustering performance in the Poisson setting.

<u>Time and Memory Scaling Analysis of ZPs and PCA</u>

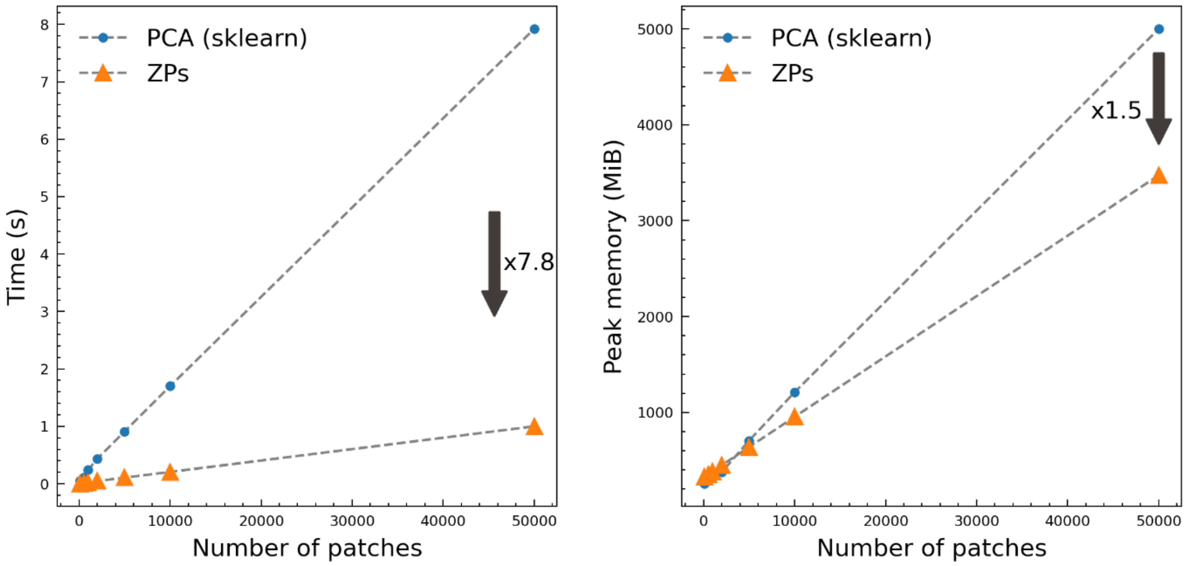

Fig. S9. Time and space time complexity analysis of PCA and ZPs.

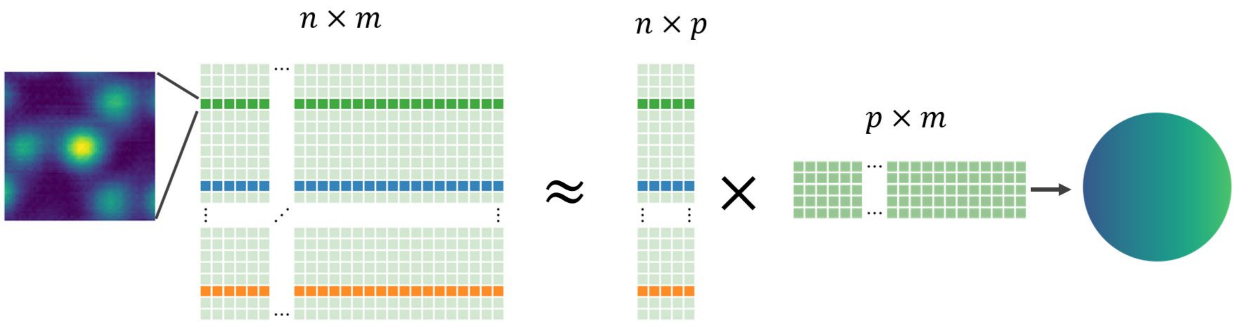

Fig. S10. Computation of Zernike moments (features) via matrix approximation. We used matrix approximation to compute Zernike features with a reduced dimension of p. A total number of n image patches was flattened to form a matrix with a shape of (n, m), where m is the number of pixels in one patch. A set of p Zernike polynomials with the same shape of image patch is also flattened to form a matrix with a shape of (p, m). The reduced features with a shape of (n, p) can be approximated via matrix pseudo-inverse operation.

Computation Zernike moments can be greatly sped up via matrix approximation instead of directly integrating. As illustrated in Fig. S10, the linear decomposition property of ZPs entitles us to simplify the computation through a matrix pseudo inverse operation. Fig. S9 shows that computation of Zernike moments via matrix approximation is about 7.8 times faster and 1.5 times more memory efficient than PCA (scikit-learn implementation).

Force-relaxed Clustering

1. Basic Definitions

Let $X = \{x_1, x_2, x_3, \cdots, x_n\}$ be a set of n features such that $x_n \subseteq R^m$. In this work, x_n represents the m Zernike moments extracted from the nth image patch. The set of such features X then suffer a second low dimensional transformation $T: X \mapsto Y = \{y_1, y_2, y_3, \cdots, y_n | \, y_n \subseteq R^d\}$, and $d \ll m$. Here, we use principal component analysis (PCA) with $d = 2$ to generate the initial layout $X \rightarrow Y$. Different low dimensional transformations can also be adopted.

The neighborhood information of X can be stored in a weighted graph $G_X = (V_X, E_X)$, where V_X are vertices (features) and E_X are edges (between valid pairs of features) of the graph. Edges are drawn between two vertices only if they have a non-zero element in their adjacency matrix, $P \in R^{n \times n}$ that we define below. The element P_{ij} ($1 \leq i \leq n$ and $1 \leq j \leq n$) of the adjacency matrix P is the weight of the edge (i, j), which measures the similarity between vertices associated with features x_i and x_j.

An isomorphic graph can be constructed with the reduced features Y as vertices: $G_Y = (V_Y, E_X)$. Notice that both graphs G_X and G_Y share the same set of edges.

2. Construction of an Adjacency Matrix P from X Using k-Nearest Neighbor Method

Given a set of vertices V_X, there are many ways to construct the edges of graph $G_X(V_X, E_X)$. In this work, we use the k-nearest neighbor method (k-NN).

Given an input hyperparameter k, we are able to compute the set of k-nearest neighbors of x_i as $\{x_i^1, \dots, x_i^k\}$. For each x_i, we define a minimum distance r_i and a normalization distance σ_i from the neighborhood. The minimum distance

$$r_i = min\{d(x_i, x_i^\kappa) | 1 \leq \kappa \leq k\} ,$$

where $d(x_i, x_i^\kappa)$ is a default distance metric between two features x_i and x_i^κ. In this work, d between two features vectors u and v is defined as

$$d(u, v) = 1 - \frac{(u - u^{-}) \cdot (v - v^{-})}{\| (u - u^{-}) \| \| (v - v^{-}) \|} ,$$

where u^{-} and v^{-} are the average of the components of feature vectors u and v respectively.

We then choose to normalize the distance metric around different features to homogenize the density of features in R^m. This choice, empirically, will cause the features to cluster at approximately similar rates. Specifically, we normalize the distance around each feature x_i with a σ_i defined in the equation:

$$\sum_{\kappa=1}^{k} \quad exp\left(-\frac{d(x_i, x_i^{\kappa}) - r_i}{\sigma_i}\right) = log_2(k).$$

Here σ_i is numerically determined using a binary search for the k-neighbors around each feature vector x_i.

Then we can construct an asymmetric adjacency matrix Q between all pairs of features.

$$Q_{ij} = \{exp\left(-\frac{d(x_i, x_j) - r_i}{\sigma_i}\right) \; if \; x_j \in \{x_i^1, \ldots, x_i^k\} \; 0 \; otherwise \,,$$

where $1 \leq i \leq n$ and $1 \leq j \leq n$.

In this notation, we can rewrite Q_{ij} as,

$$Q_{ij} = \{exp\left(-\frac{d(x_i, x_j) - r_i}{\sigma_i}\right) \; (i,j) \in E_X \; 0 \; otherwise \,.$$

The full adjacency matrix P is obtained by symmetrizing Q:

$$P = Q + Q^{\top}.$$

Edges are defined $(i,j) \in E_X$ for the graph G_X only if $P_{ij} \neq 0$. The set of all possible edges complementary to E_X is denoted by $E^{-}{}_X$. This complementary set will be used later in the repulsion stage of our clustering algorithm.

3. Updating Y According to the Neighborhood Information Stored in P

To update the features in their reduced space $Y = \{y_1, y_2, y_3, \cdots, y_n\}$, we apply attractive forces between vertices $(i,j) \in E_X$ and repulsive forces between vertices in the complementary $(i,j) \in E^{-}{}_X$. This update is done iteratively, labeled by the iteration index t, such that $1 \leq t \leq t_{max}$. Further, these force-directed updates in Y are separated into two stages, marked by iteration number $\tilde{t}$: an attraction-dominated stage ($1 \leq t \leq \tilde{t}$), followed by a repulsion-dominated stage ($\tilde{t} < t \leq t_{max}$). This iteration partition $\tilde{t}$ can be tuned by hand; here, we set $\tilde{t} = t_{max}/2$.

Additionally, we also introduce a function $\gamma(t)$ that linearly relaxes the forces from unity to zero during the attraction-dominated and repulsion-dominated stages separately (see Figure S11).

Let us denote the forces between any pair of vertices, y_i and y_j, as $f_a(||y_i - y_j||)$ for attractive forces, and $f_r(||y_i - y_j||)$ for repulsive forces. We can update the reduced features from iteration (t), $Y^{(t)} = \left\{y_1^{(t)}, y_2^{(t)}, y_3^{(t)}, \cdots, y_n^{(t)}\right\}$, to $(t+1)$ using the following recipe.

1) Given a particular ordering of the edge lists E_X, we sequentially attract the pairs of vertices $(i,k) \in E_X$ (e.g. $(1,2),(3,4),(2,1),\ldots$). Explicitly, we move each attractive pair $(i,k) \in E_X$ symmetrically,

$$y_i^{(t+1)} = y_i^{(t)} - \gamma(t) P_{ik} f_a(\| y_i - y_k \|)\left(y_i^{(t)} - y_k^{(t)}\right) y_k^{(t+1)}$$
$$= y_k^{(t)} - \gamma(t) P_{ik} f_a(\| y_i - y_k \|)\left(y_k^{(t)} - y_i^{(t)}\right).$$

2) After each attraction between two vertices (i,k), we then repel the ith vertex against 10 randomly selected non-neighbor j vertices (i.e. $(i,j) \in E^{-}{}_X$). This repulsion is also done symmetrically on $(i,j) \in E^{-}{}_X$,

$$y_i^{(t+1)} = y_i^{(t)} + \gamma(t) P_{ij} f_r(\| y_i - y_j \|)\left(y_i^{(t)} - y_j^{(t)}\right) y_j^{(t+1)}$$
$$= y_j^{(t)} + \gamma(t) P_{ij} f_r(\| y_i - y_j \|)\left(y_j^{(t)} - y_i^{(t)}\right).$$

During the attraction-dominated stage ($1 \le t \le \tilde{t}$), the customized attractive and repulsive forces $f_a(.)$ and $f_r(.)$ are respectively:

$$f_a = \frac{\alpha}{1 + \| y_i - y_j \|^N} \quad f_r = \frac{\beta}{1 + \| y_i - y_j \|^M}.$$

Then when we switch over to the repulsion-dominated stage ($\tilde{t} < t \le t_{max}$), the parameters in these forces are replaced with their tilde version (i.e. $\alpha \to \tilde{\alpha}$, $\beta \to \tilde{\beta}$, $N \to \widetilde{N}$, $M \to \widetilde{M}$).

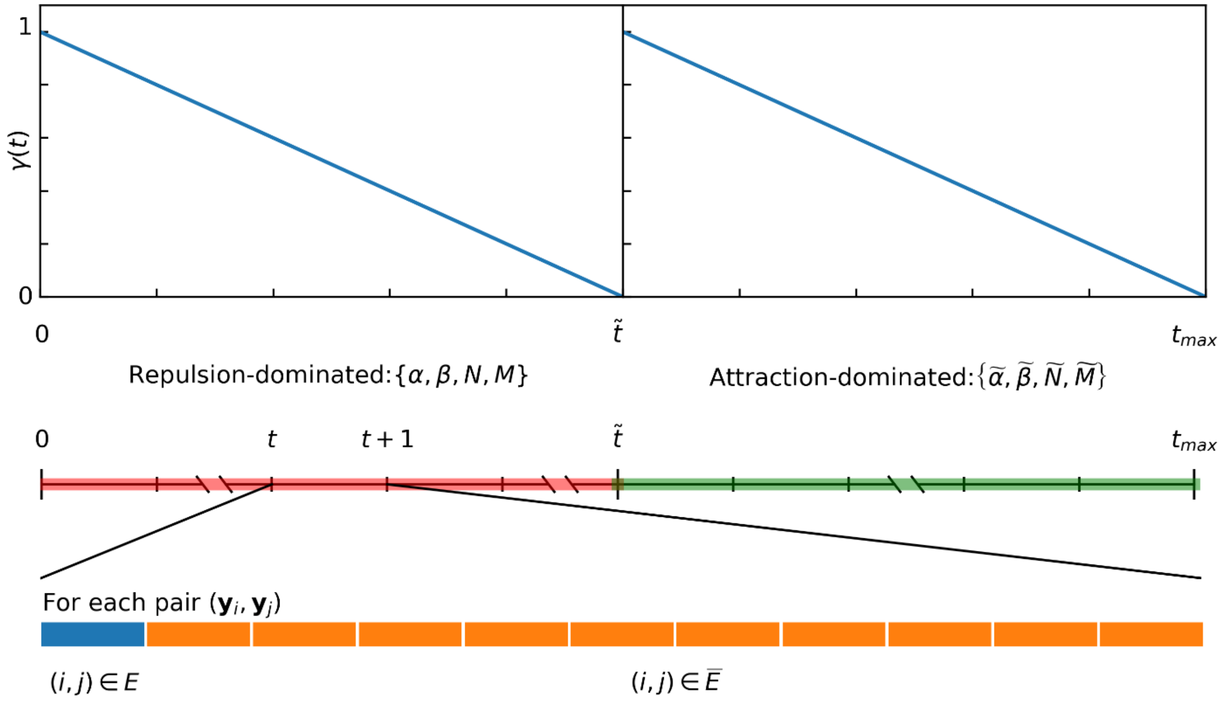

Fig S11. The schematic illustration of how forces are implemented during the two stages of the iterative relaxed clustering. The red and green color bands show the repulsion-dominated and attraction-dominated stages respectively. Consider the iteration $Y(t) \to Y(t+1)$, for each feature

$y_i \in Y$ we apply the attractive forces between $y_i$and its k-neighbors of the features (blue sub-bands), then apply repulsive forces between y_i and randomly selected non-neighbors (orange sub-bands). The displacement of force relaxation is tuned by $\gamma(t)$, which starts from 1 in each stage, then linearly falls to nearly zero at the end of the stage.

4. Deriving the Gradient of UMAP Cost Function

UMAP introduces two fuzzy sets of input (X) and output data (Y), with two sets of weights being equivalent to v_{ij} and w_{ij} respectively. The divergence function for UMAP is denoted as the cross entropy of the two fuzzy sets:

$$f_{UMAP} = \sum_{ij} \left[v_{ij} log\left(\frac{v_{ij}}{w_{ij}}\right) + (1 - v_{ij}) log\left(\frac{1 - v_{ij}}{1 - w_{ij}}\right) \right].$$

Here, the input weights v_{ij} are pre-calculated from X and treated as constants. The output weights are given by,

$$w_{ij} = 1/\left(1 + a d_{ij}^{2b}\right),$$

where $d_{ij} = |y_i - y_j|$, $d_{ij} = d_{ji}$.

The gradient of the objective function with respect to y is:

$$\frac{\partial f_{UMAP}}{\partial y_i} = 4 \sum_{j}^{N} \left[abd_{ij}^{2(b-1)} v_{ij} / \left(1 + a d_{ij}^{2b}\right) - \frac{b(1 - v_{ij})}{d_{ij}^2 \left(1 + a d_{ij}^{2b}\right)} \right] (y_i - y_j)$$

The two terms within the brackets in the last equation can be interpreted as attractive and repulsive forces acting on the features y_i respectively.

Comparison of FR, t-SNE and UMAP

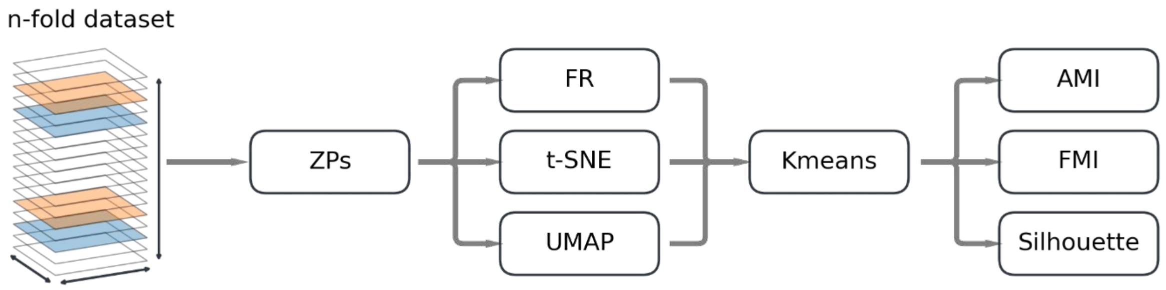

Fig. S12. A workflow to compare t-SNE, UMAP and FR. The synthetic patches consisting of two classes are represented by ZPs and embedded by t-SNE, UMAP and FR into a two-dimensional space. Kmean algorithm is applied to the corresponding 2D layout and AMI, FMI and Silhouette scores are evaluated.

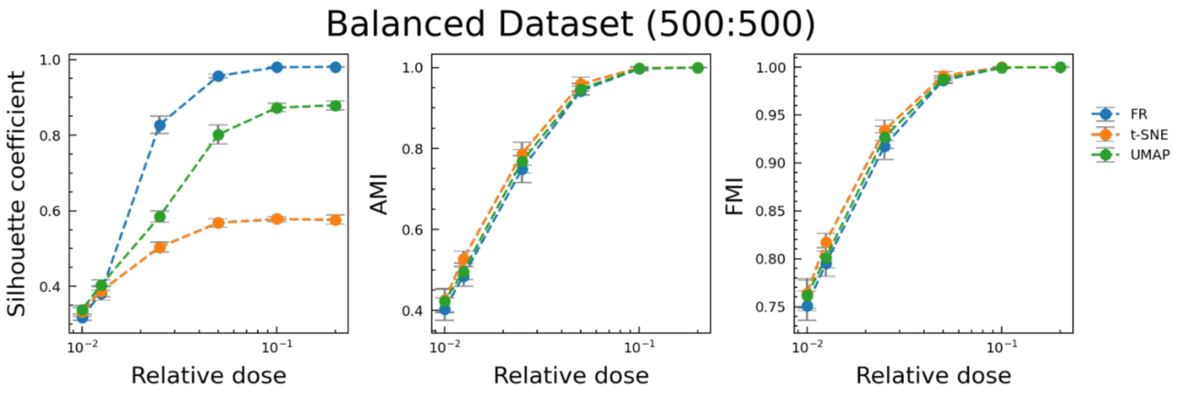

Fig. S13. Comparison of FR, t-SNE and UMAP in the synthetic balanced dataset. From left to right: Silhouette coefficients, AMI, and FMI scores variation in the presence of Poisson noises.

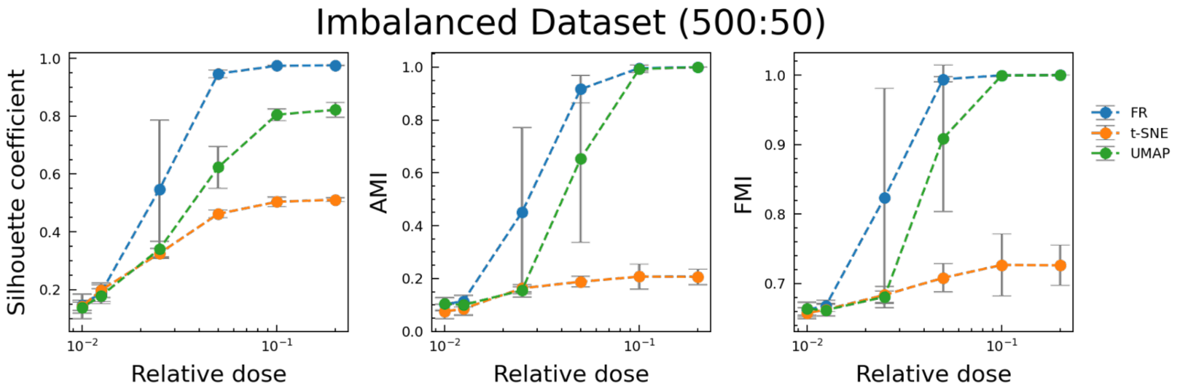

Fig. S14. Comparison of FR, t-SNE and UMAP in the synthetic imbalanced dataset. From left to right: Silhouette coefficients, AMI, and FMI scores variation in the presence of Poisson noises.

We used clustering performance scores to evaluate and compare FR, t-SNE and UMAP. Different from a classification task, evaluating clustering performance is not as trivial as computing the precision and recall according to the labels. Specifically in the context of clustering, our metric should take the cluster separation into consideration rather than counting the absolute values of

predicted labels. Similar to comparing different representation schemes (Fig. S8), we used Silhouette coefficient, AMI and FMI to evaluate the performance of FR, t-SNE and UMAP (Fig. S13 and S14).

Following the workflow in Fig. S12, we found all three embedding techniques have comparable performance in the synthetic dataset with equal-sized clusters. In the uneven-sized cluster scenario, FR outperforms UMAP and t-SNE in Silhouette, AMI, and FMI scores.

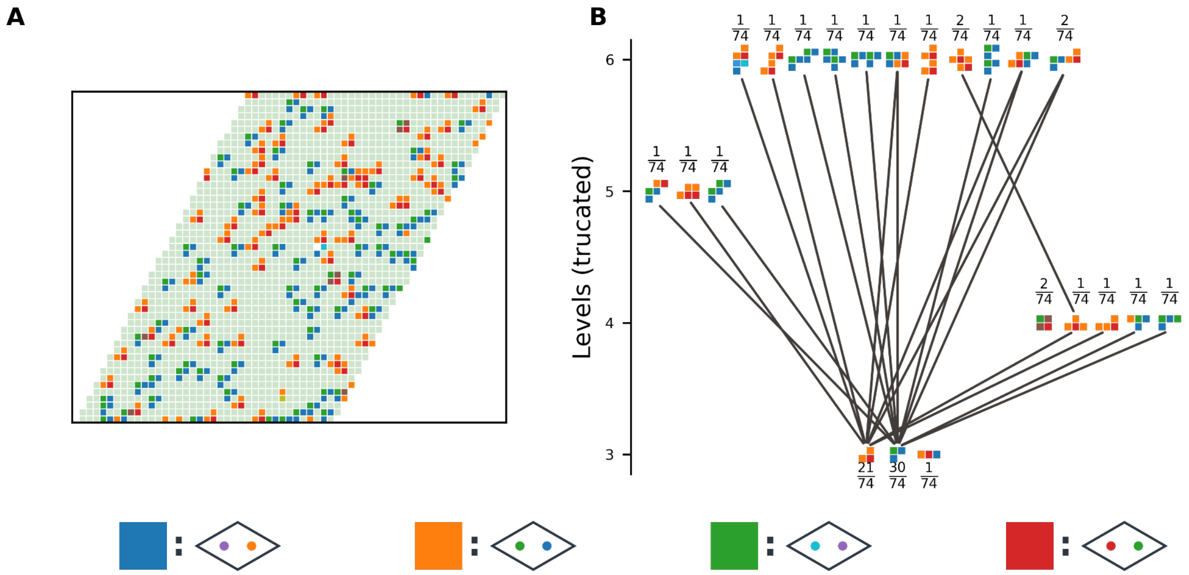

Fig. S15. Construct a motif hierarchy of the WS_2 sample. (A) Mapping realspace motif cells into a square grid. These motif-cells are colored according to the motif composition. (block legends below illustrate their realspace correspondents). **(B)** Motif-cells in (A) are ordered according to the number of cells. We associate higher-level motif-cells with lower-level ones if the spatial arrangement of cells in the latter occur within the former; here edges are drawn between associated motif-cells that are the nearest in the hierarchy.

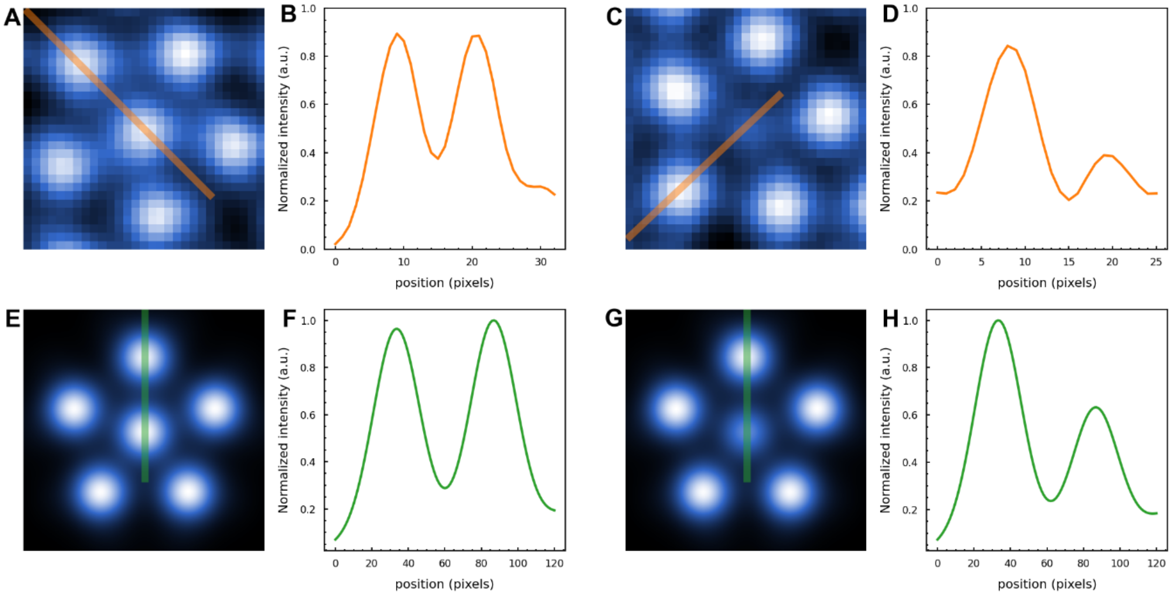

Fig. S16. Line profile analysis of filled and partially filled pentagonal units in Figure 4 of main manuscript. (A) Average motif of type1 filled pentagon unit. (B) Line profile extracted from (A), which is consistent with Mo or Ni centered pentagons. (C) Average motif of type 2 filled pentagon unit. (D) Line profile extracted from (C), which is consistent with V-centered motifs. (E) Simulation of filled pentagon unit. (F) Line profile extracted from (E). (G) Simulation of V filled pentagons unit. (H) Line profile extracted from (G).

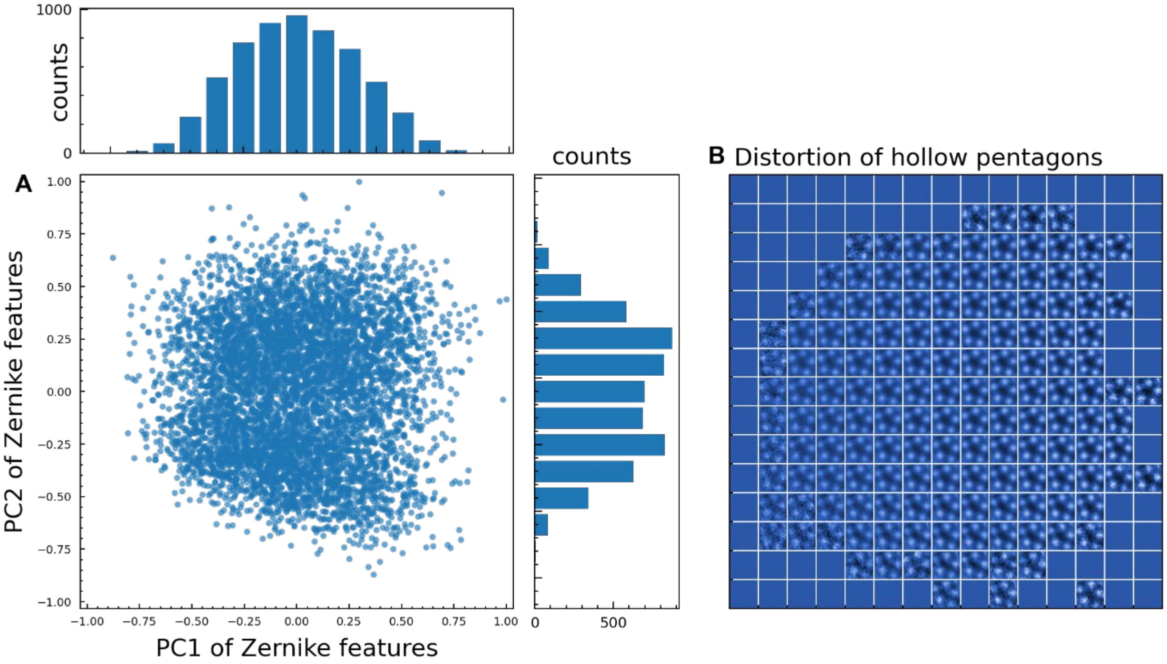

Fig. S17. Distortion mapping of hollow pentagon units. (A) PCA of rotational invariant Zernike features of hollow pentagon motifs. (B) Motif embedding showing the distortion of these hollow pentagon units.

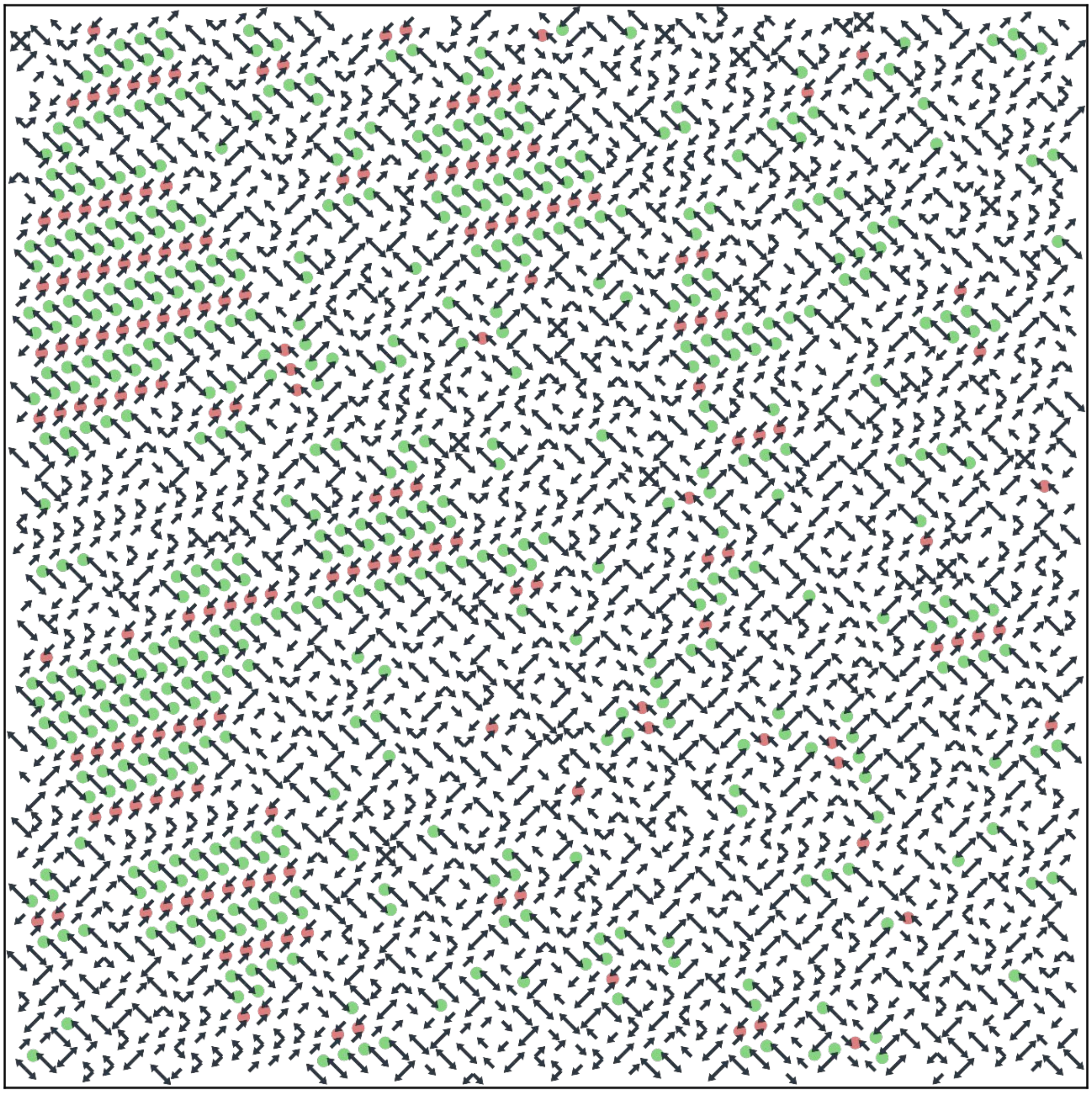

Fig. S18. Reconstruction of the ADF-STEM image with arrows from second level motifs.

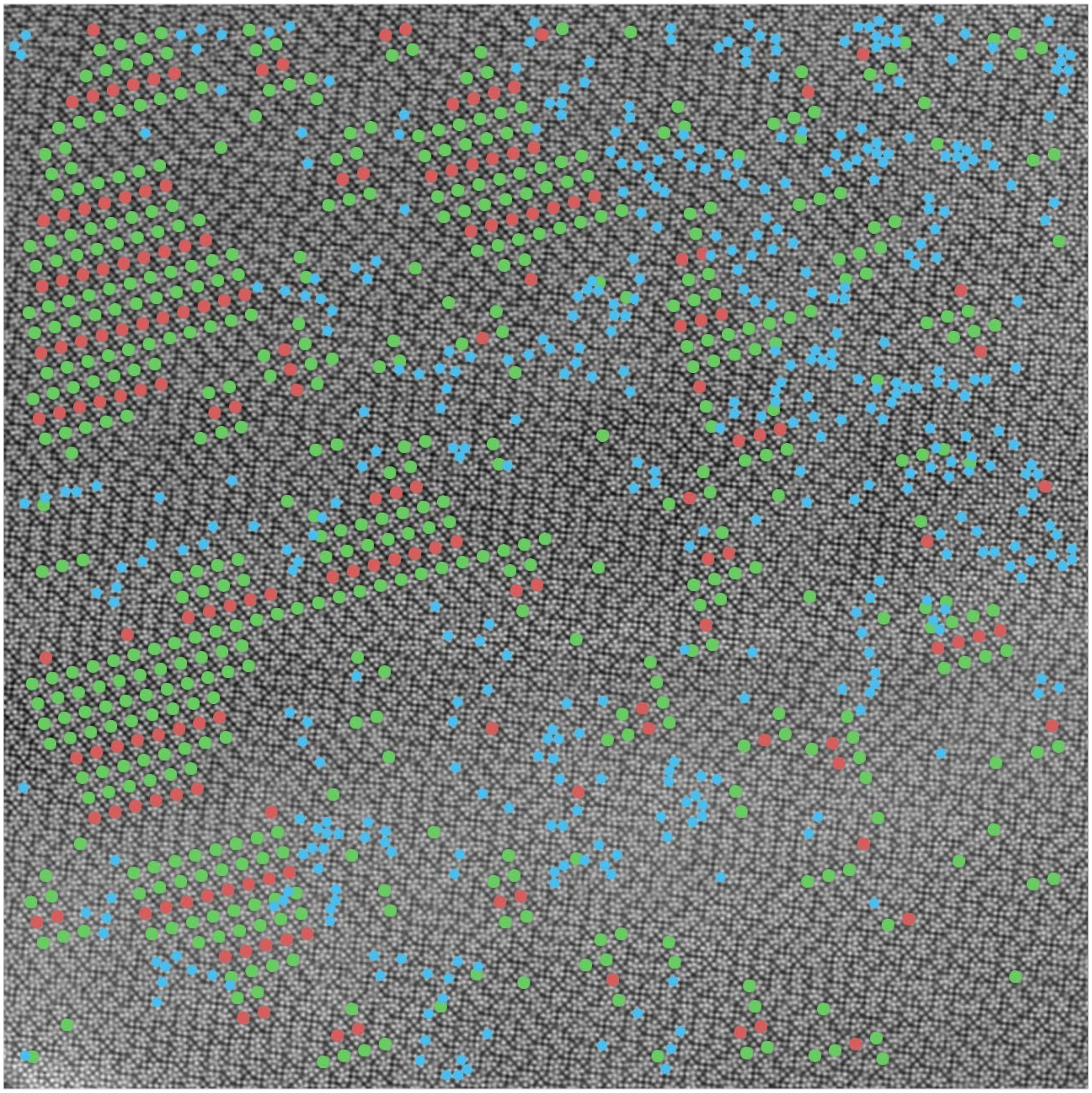

Fig. S19. Largest dominant motifs clearly show a novel phase that could tessellate the plane, but are frustrated by other competing structural motifs.

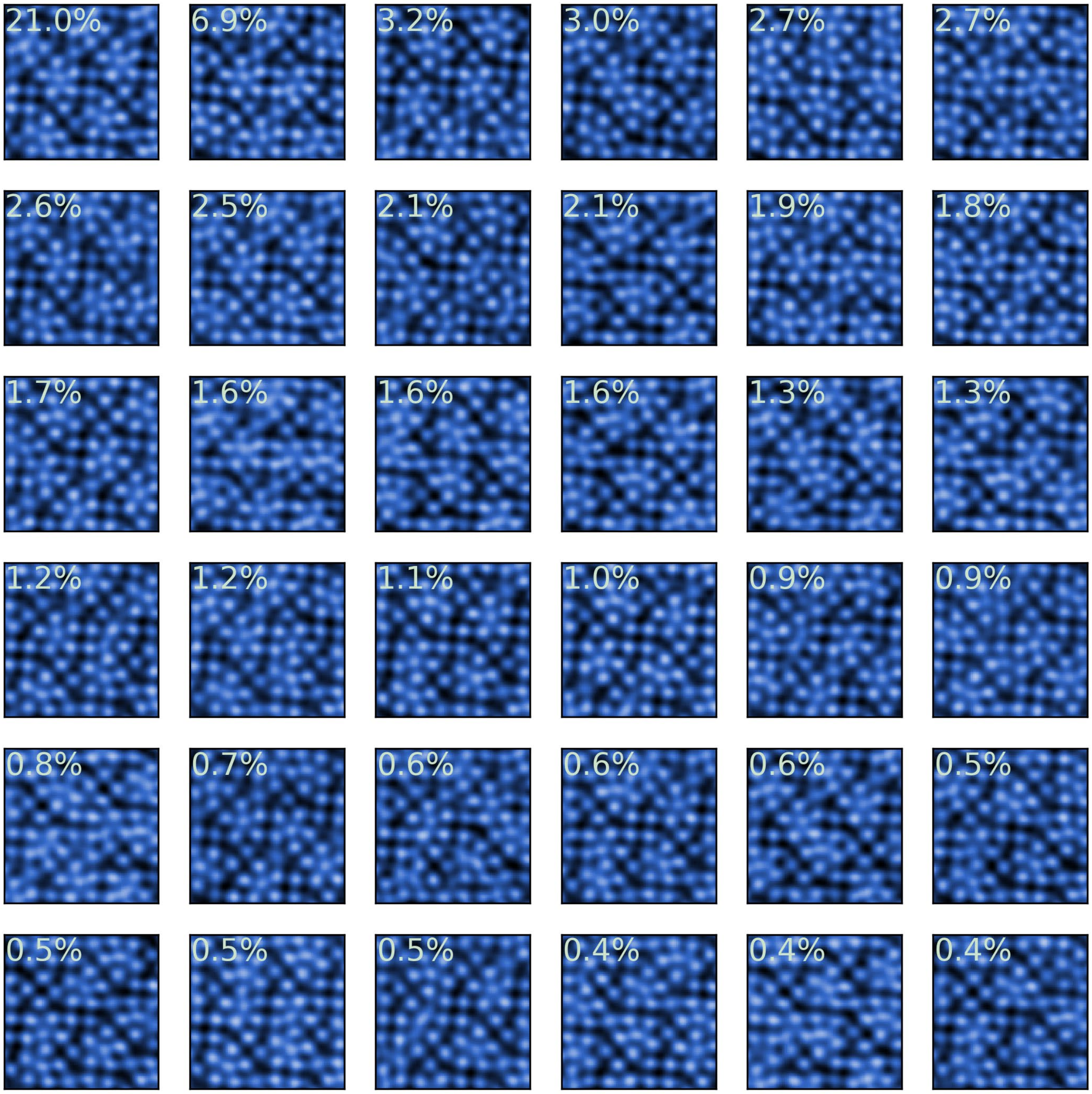

Fig. S20. Top 36 dominant third level motifs in the Mo-V-Te-Nb-oxide POM. They account for 74% of total third level motifs. The information entropy computed from all level 3 motifs via $\sum p_i log\,(p_i)$ is 4.13, where p_i is fraction of ith motif. The maximum information entropy in a completely disordered sample should be $log\,(2^{16}) \approx 11.09$.

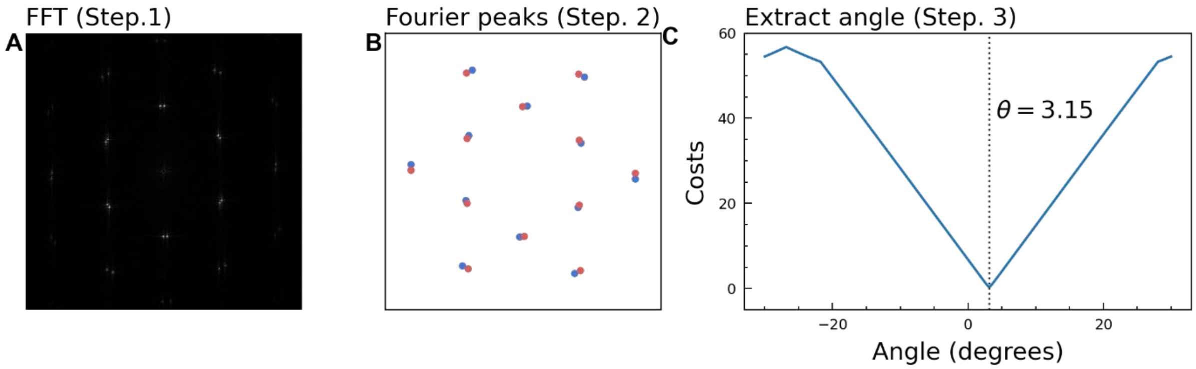

Fig. S21. Workflow for extracting relative angle in bilayer MoS_2. (A) the Fourier transform of ADF-STEM image of bilayer MoS_2. (B) Extracted two sets of peaks belonging to each layer of the moiré pattern. (C) By rotating one set of peaks in (B) and calculating the Euclidean distances mean to the other set of the peaks. The minimum value corresponds $\theta = 3.1$.

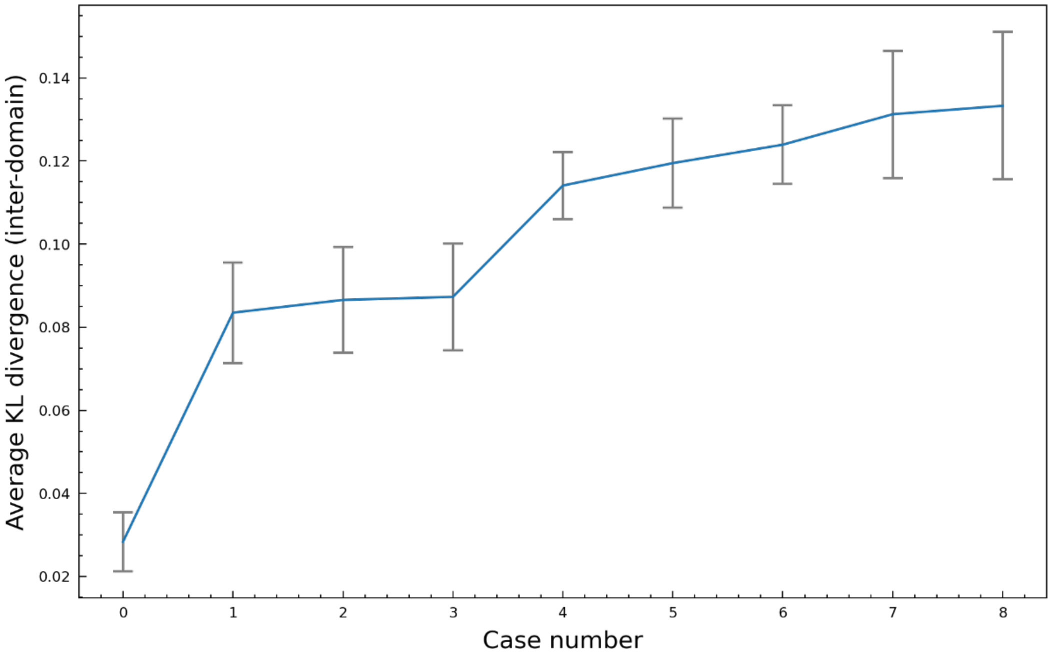

Fig. S22. Evaluation of Kullback-Leibler (KL) divergences of different domains in various cases of synthetic datasets. Synthetic data is generated by adding two misaligned layers (with relative angle θ) of hexagonal atomic columns which are modeled by two sets of Gaussian functions with relative intensity ratio r and standard deviation σ (also see Materials and Methods). Case 0: commensurate lattice $\theta = 3.14965734$. Case 1: incommensurate case $\theta = 3.2$. Case 2: increase the relative intensity ratio from 0.5 (case 1) to 0.8. Case 3: increase the size of Gaussian blobs from case 2($\sigma = \frac{a}{8} \rightarrow \sigma = \frac{a}{6}$). Case 4: add white noise ($\sigma_{noise} = 0.05$) to case 3. Cases 5 to 8: add random positional disorders to case 4 with maximum jump length setting to 0.02a, 0.05a, 0.1a and 0.15a respectively, where a is the lattice constant of the hexagonal single layer of synthetic data.

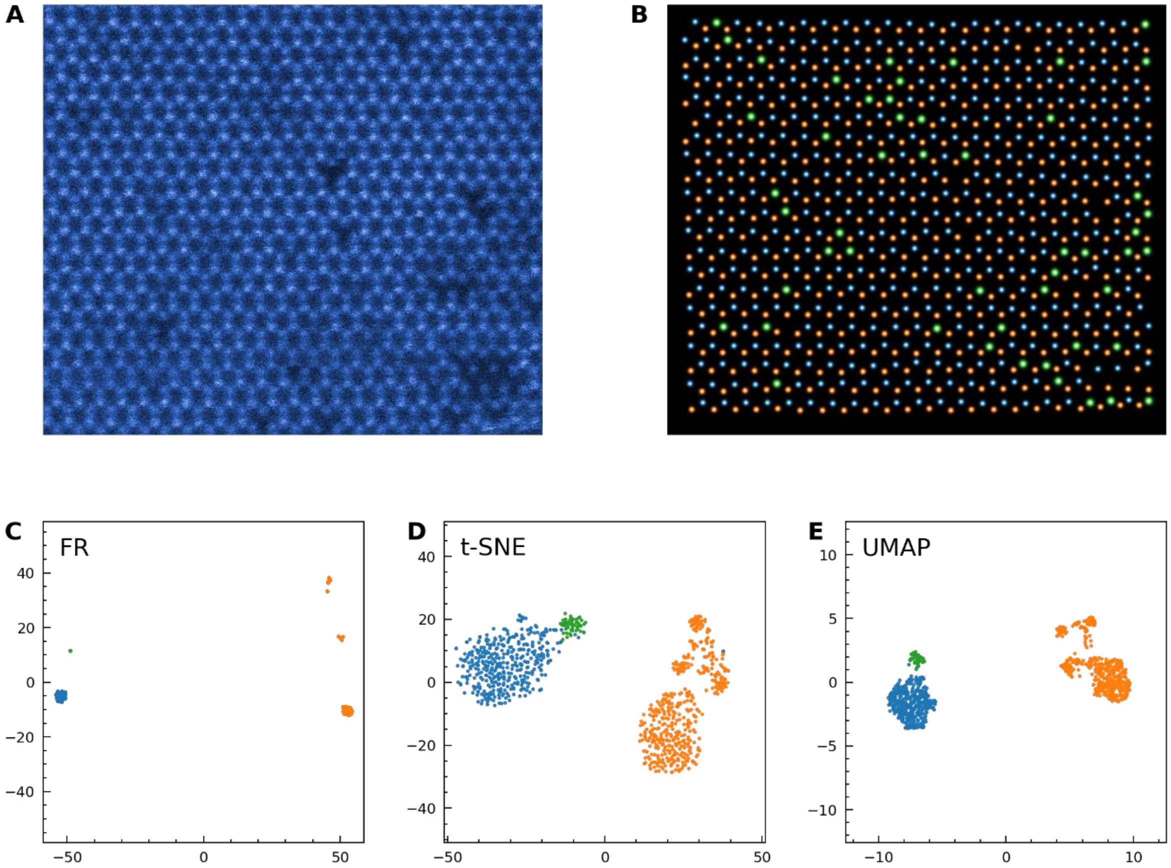

Fig. S23 Identification of Se vacancies in a low SNR STEM image of monolayer $MoSe_2$ imaged with 40 kV electrons. (a) ADF-STEM image of $MoSe_2$ with vacancy sites. **(b)** Identification map of Mo columns (orange), Se_2 columns (blue), and single Se vacancy columns (green) from **(a)**. **(c)** Cluster map using the two-stage relaxed clustering scheme (this work). **(d)** Cluster map obtained from the t-SNE algorithm. **(e)** Cluster map obtained from the UMAP algorithm.

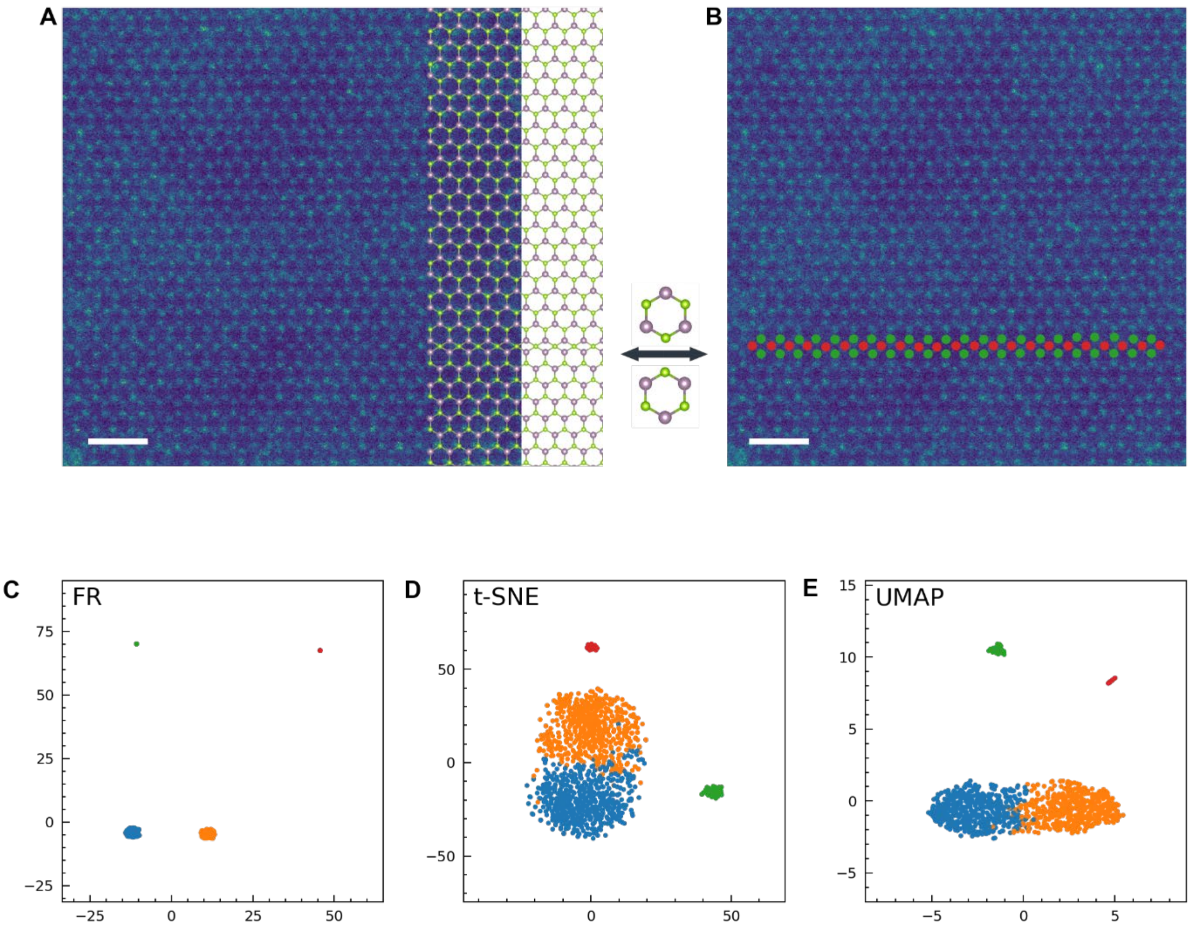

Fig. S24 Identification of a mirror twin grain boundary (MTB) in low signal-to-noise ratio STEM image of monolayer $MoSe_2$. **(A)** ADF-STEM image of monolayer $MoSe_2$ with MTB. **(B)** Identification of the grain boundary. The atomic columns indicated in green and red dots correspond to clusters of the same color in **(C)**. **(C)** Cluster map obtained from the proposed two-stage relaxed clustering algorithm (k=10, k'=5). **(D)** Cluster map obtained from the UMAP algorithm. **(E)** Cluster map obtained from the t-SNE algorithm. The blue and orange clusters represent Mo columns and Se_2 columns respectively. (scale bar: 1nm)

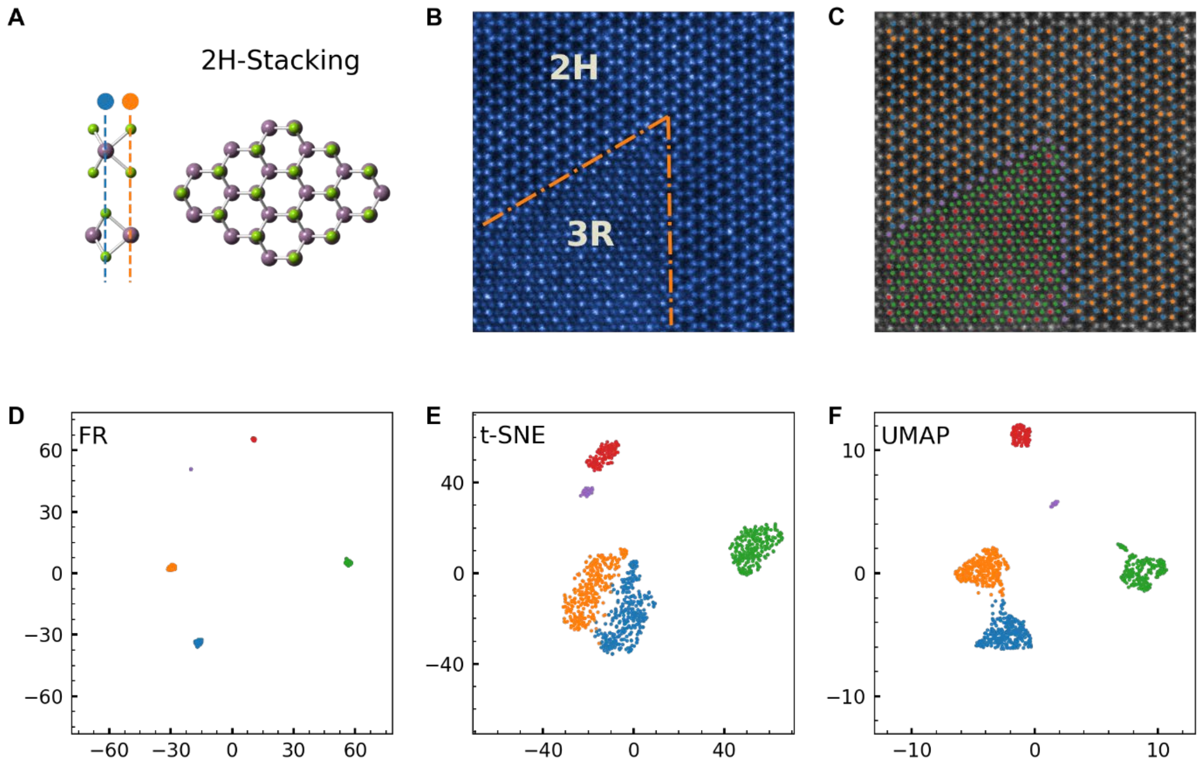

Fig. S25 Identification of 2H and 3R phases and their phase boundaries in a STEM image of bilayer $MoSe_2$. **(a)** Schematic model of 2H stacking. **(b)** ADF-STEM image of bilayer $MoSe_2$ with the 2H-3R boundaries indicated by orange dashed lines. **(c)** Identification map from the proposed method. **(d)** Cluster map obtained from the proposed two-stage relaxed clustering method (k=10, k'=5). **(e)** Cluster map obtained from t-SNE. **(f)** Cluster map obtained from UMAP.

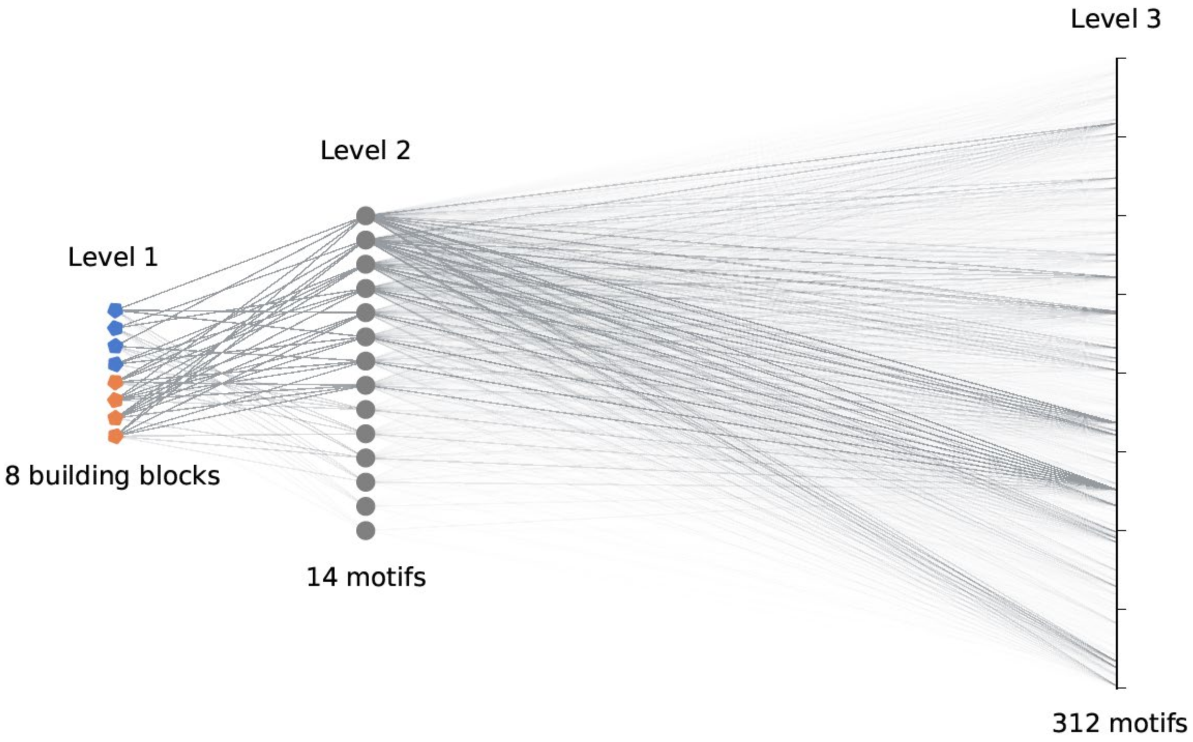

Fig. S26 Complete motif hierarchy for Mo-V-Te-Nb-oxide POM.

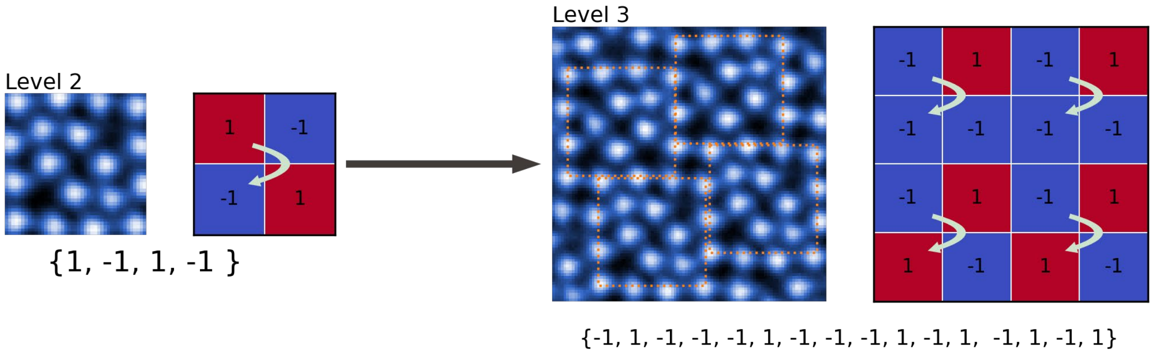

Fig. S27. Encoding of level 2 and level 3 motifs using binary values of -1 and 1. -1 denotes the hollow pentagon and 1 represents filled pentagon. Level 2 motifs can be encoded as a vector of length 4, and level 3 motifs can be represented by concatenating level 2 motif vectors, resulting in a vector of length 16.

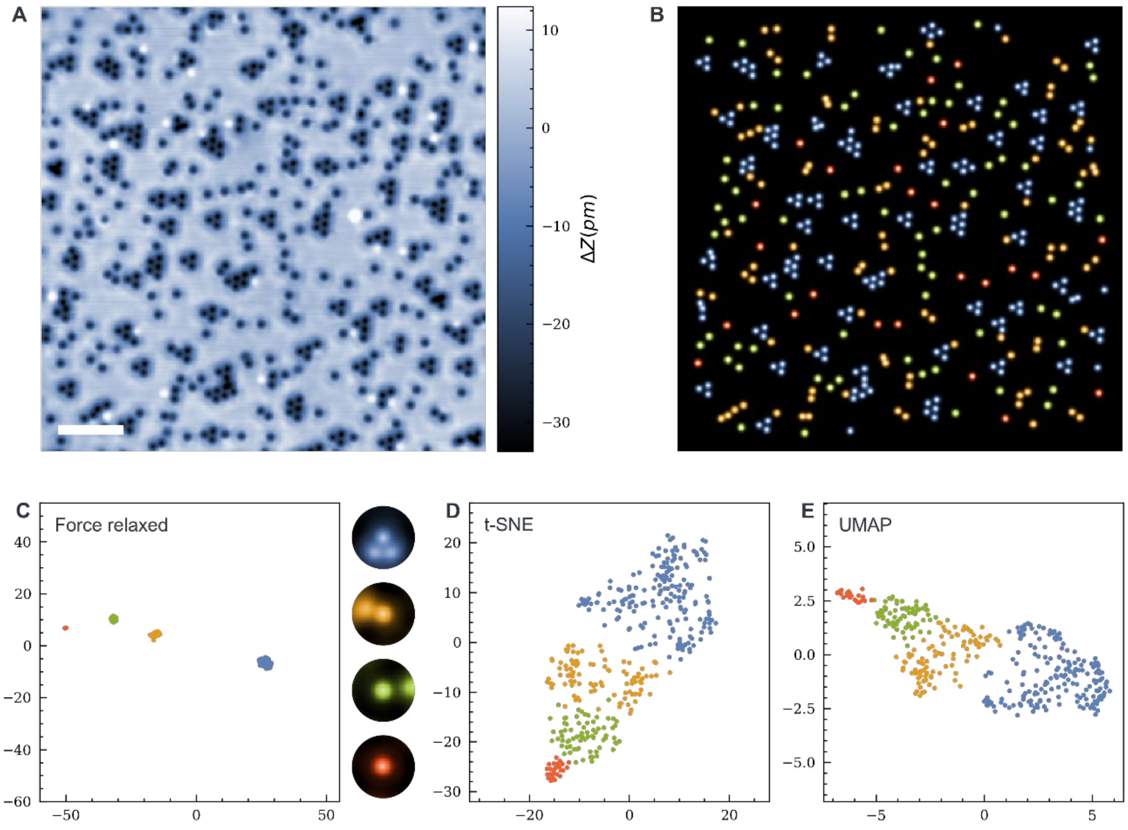

Fig. S28. Comparison of clustering results using different low dimensional embedding methods in an STM image of a $PtTe_2$ thin film. **(A)** STM image of $PtTe_2$ with Te vacancy sites (collected at sample bias Vs=-2 V and tunneling current I=300 pA) **(B)** Cluster map of all vacancy sites in **(A)**. Red and blue dots correspond to isolated vacancies and clusters, while green and orange represent chains of different lengths. **(C)** Cluster map using a two-stage force-relaxation clustering scheme. **(D)** Cluster map obtained from the UMAP algorithm. **(E)** Cluster map obtained from the t-SNE algorithm. Scale bar: 5 nm in (A).

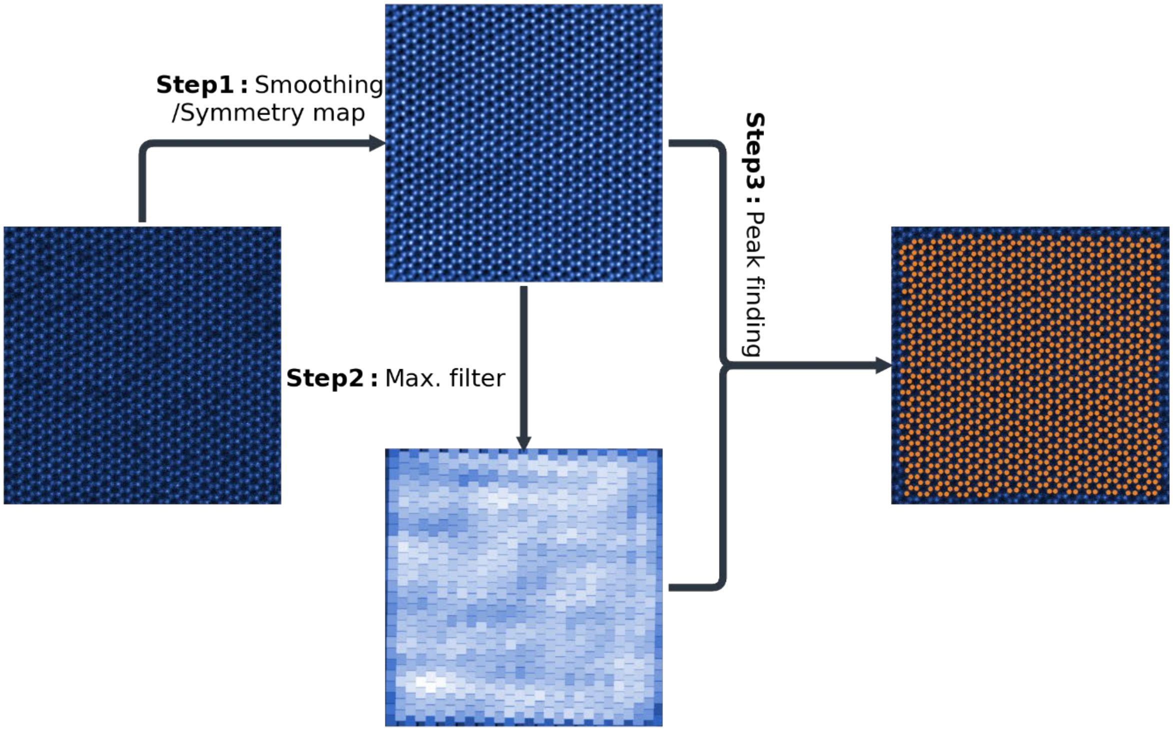

Fig. S29. Workflow to locate the position of feature points.

Fig. S29 illustrates a three-step workflow to locate feature points. The key to successfully extracting feature points is to obtain smooth versions of raw images (**Step 1**). Depending on image quality and image conditions, different smoothing schemes are adopted. For images with a high signal-to-noise ratio (SNR), Fourier space filtering was implemented to keep 10% of the lowest frequency components. For images with low SNR, Singular Value Decomposition based method was applied to the image. We adopted the SVD based method for images in this work unless stated otherwise. The smooth image was dilated by a local maximum filter (**Step 2**). The feature points are the locations where the input image is equivalent to the dilated version (**Step 3**).

Patch position perturbation analysis

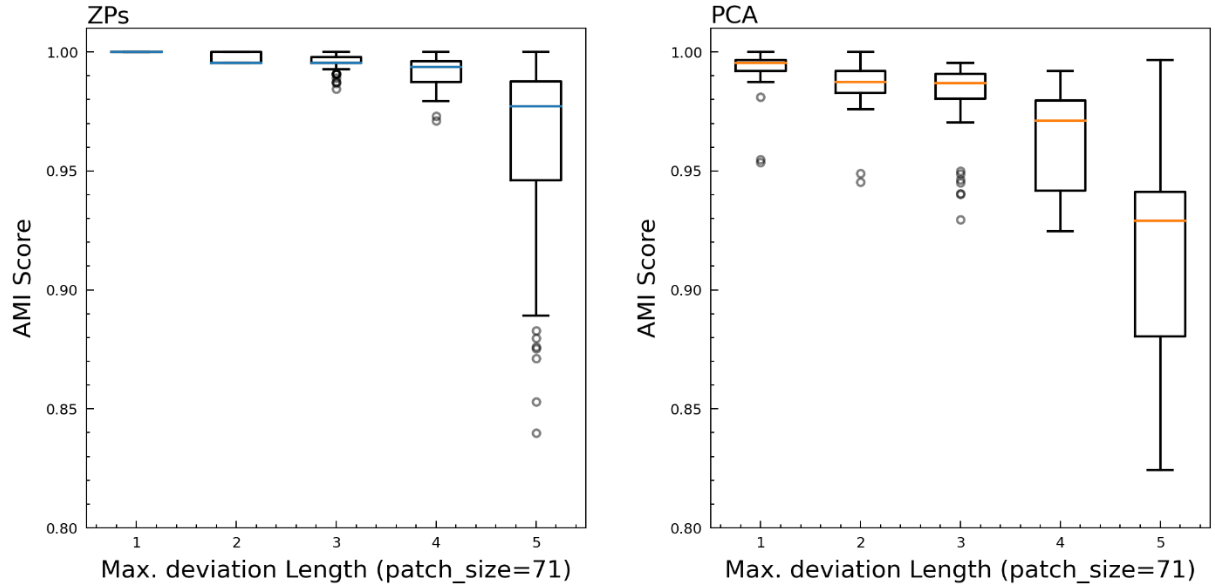

Fig. S30. Zernike representation is more robust to patch position deviation. Left panel: adjusted mutual information score vs. position deviation length when ZPs are implemented. Right panel: adjusted mutual information score vs. position deviation length when PCA is used.

The combination of Zernike representation plus FR clustering is more tolerant to patch position perturbation compared with the combination of PCA and FR.

We use our $MoSe_2$ example and adjusted mutual information (AMI) score to solidify this point. We randomly perturb positions of derived feature points by a uniform distribution bound by a maximum deviation length L (in units of pixels). When L is changed from 1 to 5 (corresponding to 0.08 - 0.40 Å in this example), our proposed workflow produces 5 sets of clustering results. The consensus measurement (*e.g.,* AMI) from the clustering of no perturbation should be close to unity if our scheme is robust. We sampled 50 AMI scores for each boxplot to get AMI vs. L using Zernike representation plus FR clustering. Moreover, when Zernike representation is replaced by PCA, it is clear that PCA is less robust to variation of patch positions from Fig. S30 (right panel).

Fig. S30 shows the change of Adjusted mutual information (AMI) vs. L, we see an obvious drop of the consensus measurement when L = 5 (0.40 Å), which is 7% of the patch size (71) in the example. This satisfies the accuracy requirement of normal peak-finding algorithms from either symmetry maps or denoised STEM images.

Workflow to determine the patch size

Empirically the side length of patches is estimated to be the radius of the first dominant peak in radius distribution function (RDF) of the underlying sample, which can be approximated from the Fourier transform of atomic resolution images. When the patch sizes are too large, the downstream FR algorithm will only capture the major clusters as small satellite clusters cannot be distinguished because they share high similarity with the corresponding major cluster; When the patch sizes are too small, the patch does not cover sufficient region and the local environment (spatial) information is lost, which make it comparable with conventionally Z-contrast method based on intensity profile.

Briefly, should the sample have a known periodicity, then the patch size should be about the same size as the repeating unit. This choice allows enough features to get discrete classes (i.e., motifs) from the FR clustering (see Figure S1). The residual disorder beyond the length scales of a single patch will then emerge in the hierarchy constructed from these motifs.

Here's our prescription for how patch sizes can be programmatically determined. When it comes to the selection of patch sizes, it is a hyperparameter that can be automatically computed via the following steps:

1. Compute the FFT of the input image (power spectrum image).

2. Obtain the average radial intensity curve from the power spectrum image and locate the radius of the first dominant peak from the average radial intensity curve.

3. Convert the radius calculated from FFT space to image (real) space.

The above process is summarized in a workflow as shown in Fig. S31.

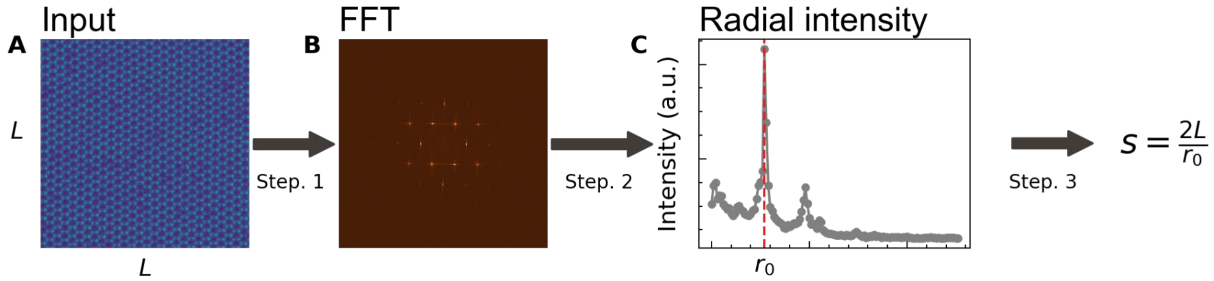

Fig. S31. Workflow to estimate the motif size for patch extractions. (A) An atomic resolution STEM image with a shape of (L, L). (B) FFT of the input image in (A). **(C)** Average radius intensity calculated from panel (B), and r_0 is the position of the first dominant peak. In the final step, the patch size is empirically calculated by $s = \frac{2L}{r_0}$.

(p, q)	(0, 0)	(1, -1)	(1, 1)	(2, -2)	(2, 0)	(2, 2)	(3, -3)	(3, -1)	(3, 1)
j	0	1	2	3	4	5	6	7	8
(p, q)	(3, 3)	(4, -4)	(4, -2)	(4, 0)	(4, 2)	(4, 4)	(5, -5)	(5, -3)	(5, -1)
j	9	10	11	12	13	14	15	16	17

Table S1. Relation between indices $(p,\ q)$ **and** j.

**FR Clustering**	
**Total iterations**	Time (s)
**10**	0.57 ± 0.05
**20**	0.70 ± 0.05
**40**	0.87 ± 0.05
**80**	1.28 ± 0.10
**160**	2.17 ± 0.12
**Hierarchy Construction**	
Step 1. Identify motif-cells	0.17 ± 0.03
Step 2. Connect motif-cells	0.10 ± 0.02
Step 3. Construct hierarchy	0.32 ± 0.01

Table S2. Computation time for FR clustering for the various number of iterations (*k*=15, *k*'=5, PCA initialization). These timing tests were performed on 1207 features from the model system monolayer $MoSe_2$ in Fig. 1A, using a single-threaded process on a desktop computer with Intel Xeon CPU E5-2630Lv3 @ 1.80 GHz and 24 GB RAM. Given the same hardware, concurrent programming paradigms will further accelerate these processing times.

	Class 1 ($\sigma = 7$)	Class 2 ($\sigma = 7$)
3-fold	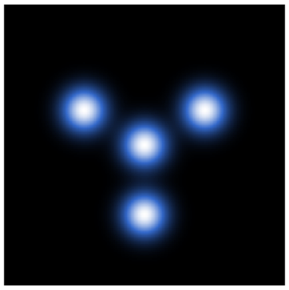	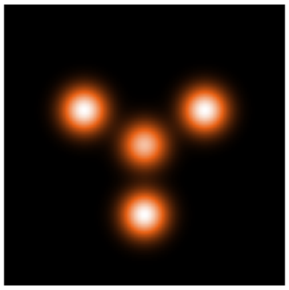
4-fold	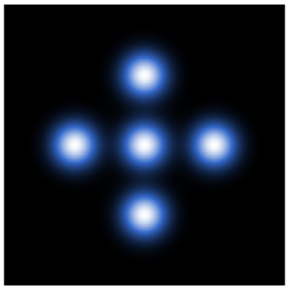	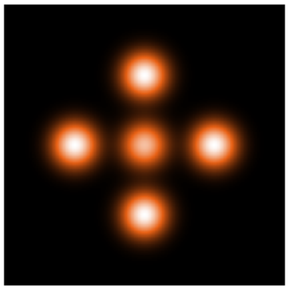
5-fold	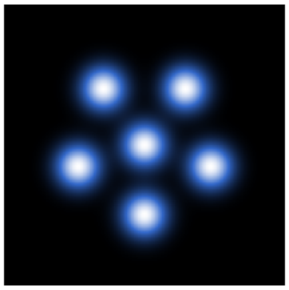	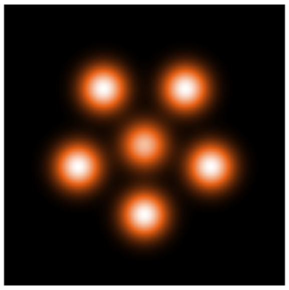
6-fold	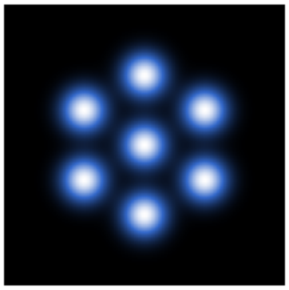	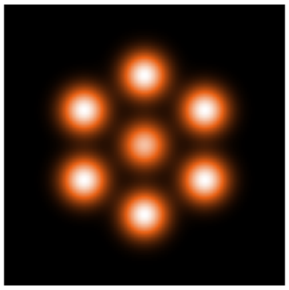
7-fold	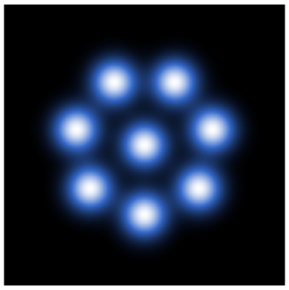	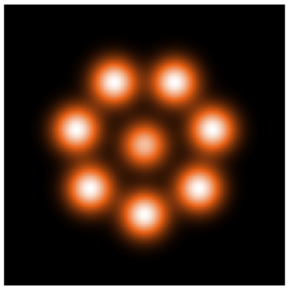

Table S3. A visual display of the synthetic dataset when default parameters are used.

Synthetic Dataset description: By default, each synthetic motif with *n*-fold rotational symmetry has a shape of 128x128 which contains (*n*+1) Gaussian blobs. These (*n*+1) Gaussian blobs form a regular n-gon with the extra Gaussian blob occupying the center. The surrounding Gaussian blobs have a distance of 32 pixels away from the center of the motif. All Gaussians share the same sigma value of 7. In all blue motif classes, all Gaussian blobs have a maximum amplitude of 1.0, while in the orange motif classes, the central blob has a maximum amplitude of 0.80. In the case of applying Poisson noise to the above data, the motif is scaled and converted to an integer type.

A synthetic dataset can be specified by a unique set of parameters $\{n1, n2, s, \sigma, l, A\}$, and the definitions are these parameters are declared as follows (default values in parentheses):

- $n1$ – the number of patches for class 1
- $n2$ – the number of patches for class 2
- s – the size of every patch ($s = 128$)
- σ – the sigma value of the Gaussian blobs contained in each patch ($\sigma = 7$)
- l – the distance of surrounding Gaussian blobs to the central blobs ($l = 32$)
- A – the relative intensity of central blobs in class 2 ($A = 0.8$)

	**Attraction force**	**Repulsion force**	**Remarks**
LargeVis	$\frac{2}{(1+d^2)}$	$\frac{2\gamma}{(1+d^2)(d^2+\epsilon)}$	$\gamma = 7, \epsilon = 0.1$
UMAP	$\frac{2ab\, d^{2b-2}}{(1+a\, d^{2b})}$	$\frac{2\gamma b}{(1+a\, d^{2b})(d^2+\epsilon)}$	$\gamma = 1, \epsilon = 0.001$<br>$a = 1.58, b = 0.90$
**Two-stage relaxed clustering (this work)**	$\frac{\alpha}{1+d^n}$	$\frac{\beta}{1+d^m}$	$m \geq 0, n \geq 0$<br>$\alpha > 0, \beta > 0$
Here *d* refers to the distance between two PCA-reduced features: $\boldsymbol{d} = \|\|\boldsymbol{Y_u} - \boldsymbol{Y_v}\|\|$.			

Table S4. A summary of force functions in different iterative clustering algorithms

Movie S1

Cluster dynamics of a two-stage FR layout evolution. It comprises a repulsion-dominated stage (stage one), followed by an attraction-dominated stage (stage two). The repulsion-dominated stage allows adequate separation distance between structural motifs whose features are mutually most dissimilar. The attraction-dominated stage then adjusts the strength of attraction force to make sure each motif cluster is compact, and that clear decision boundaries can be drawn between clusters.
